# Supplementary material for: Sarc-Graph: Automated segmentation, tracking, and analysis of sarcomeres in hiPSC-derived cardiomyocytes
Source: PLoS Comput Biol. 2021 Oct 6;17(10):e1009443. doi: 10.1371/journal.pcbi.1009443 (PMC8523047; doi:10.1371/journal.pcbi.1009443)
Supplement: S1 Text — Direct comparison to an alternative method for automatically quantifying morphology of single images. Fig A. Pre-processing with a Gaussian filter. The effect of applying a Gaussian filter as a pre-processing step on Sarc-Graph illustrated on data from Sutcliffe et al. 2018 [12]. Fig B. Direct comparison between Sarc-Graph and SOTA [12]. Comparison between SOTA and Sarc-Graph of sarcomere segmentation and analysis for the images shown in Figure 6 of Sutcliffe et al. 2018 [12]. (PDF) [file pcbi.1009443.s001.pdf]

Sarc-Graph: Automated segmentation, tracking, and analysis of sarcomeres in hiPSC-derived cardiomyocytes
S1 Text · Sutcliffe et al. 2018 [1] Comparison

Description of the comparison software:
SarcOmere Texture Analysis (SOTA) is introduced in the paper "High content analysis identifies unique morphological features of reprogrammed cardiomyocytes" [1]. SOTA contains many tools to analyze cell morphology, here we focus exclusively on the component of the software used to analyze sarcomeres. In particular, we focus on making a direct comparison to the SOTA automated measure of sarcomere length. The SOTA code is implemented in MATLAB, and available from `https://github.com/saucermanlab/SarcOrgTextureAnalysis`. For reference, critical key differences between SOTA and Sarc-Graph are as follows:

- To our knowledge, SOTA is used for analyzing static images and does not contain any features for tracking individual sarcomeres with respect to time. Therefore, we are only able to make a direct comparison between SOTA and the segmentation component of the Sarc-Graph framework.
- To our knowledge, SOTA is only able to provide average information over specified regions of interest.
- To our knowledge, SOTA applies a Gaussian filter with size 4 to the images prior to processing. Sarc-Graph does not. Here we investigate the influence of applying a Gaussian filter as a pre-processing step for Sarc-Graph (Fig 1) and find that it substantially influences our results.

Description of the comparison data:
The comparison dataset was published in conjunction with the SOTA paper [1]. We downloaded the dataset from this figshare url: `https://figshare.com/articles/dataset/Sutcliffe_et_al_Scientific_Reports_2018_images/11390022`. We focused specifically on the images shown in Fig 4 and Fig 6 of the original manuscript. We note briefly that we follow the conversion provided in the SOTA MATLAB script where $1\,\mu m/1$ pixel $= 160/1024$. Broadly speaking, we did not find agreement between our results and the results generated with SOTA. We believe that this discrepancy is due to the SOTA pre-processing step of applying a Gaussian filter with size 4 pixels to the images [1].

Figure A. The effect of applying a Gaussian filter as a pre-processing step on Sarc-Graph.
Here we apply Gaussian filters of different size as a pre-processing step, then segment individual sarcomeres with Sarc-Graph. Segmented z-discs are outlined in light blue, segmented sarcomeres are indicated by a red star. For the standard implementation of Sarc-Graph, we do not apply a filter as a pre-processing step (i.e., filter size = 0). For the standard implementation of SOTA, a filter of size 4 is applied as a pre-processing step.

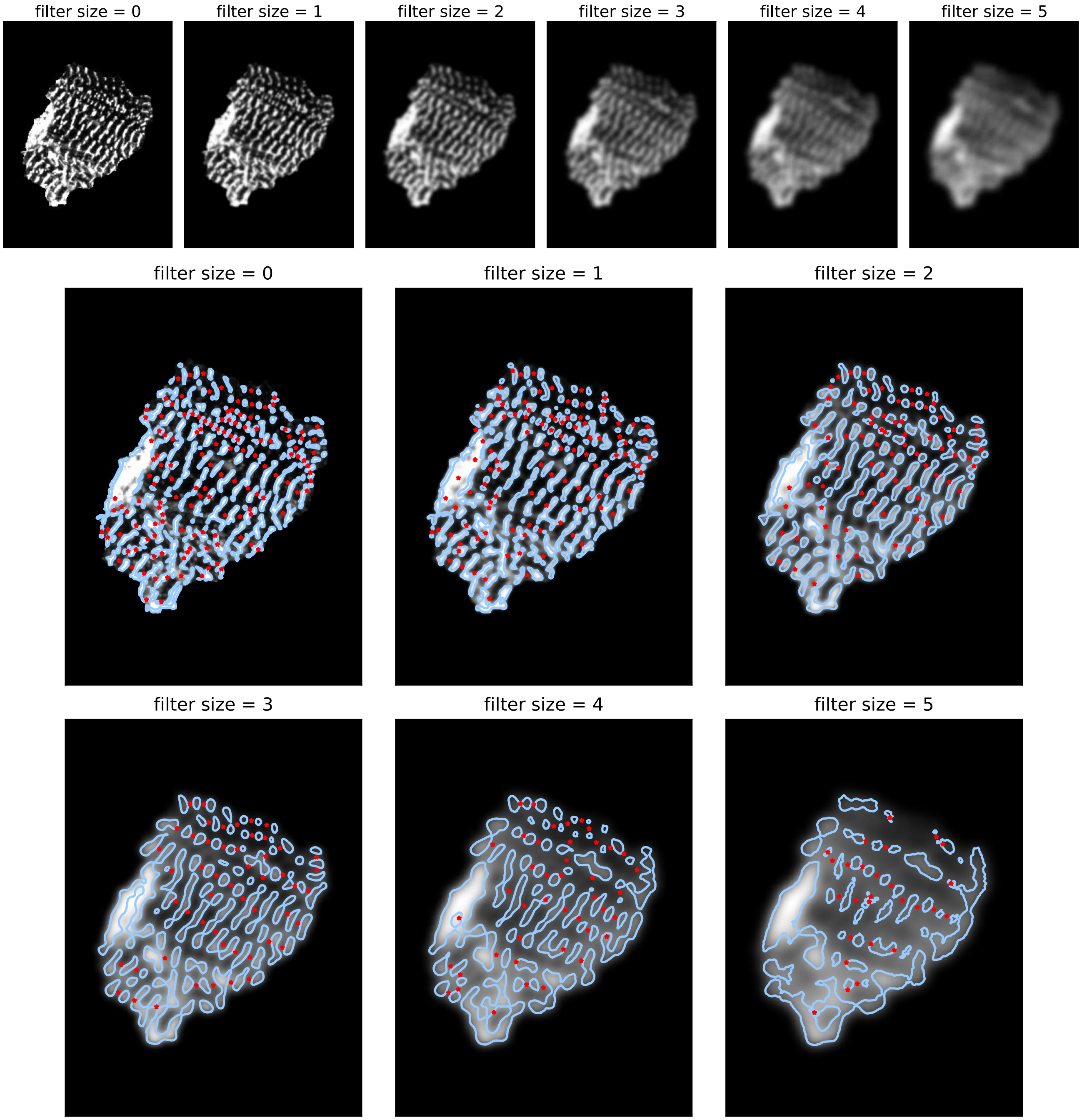

Number of sarcomeres and mean sarcomere length segmented with Sarc-Graph for different Gaussian filter sizes:

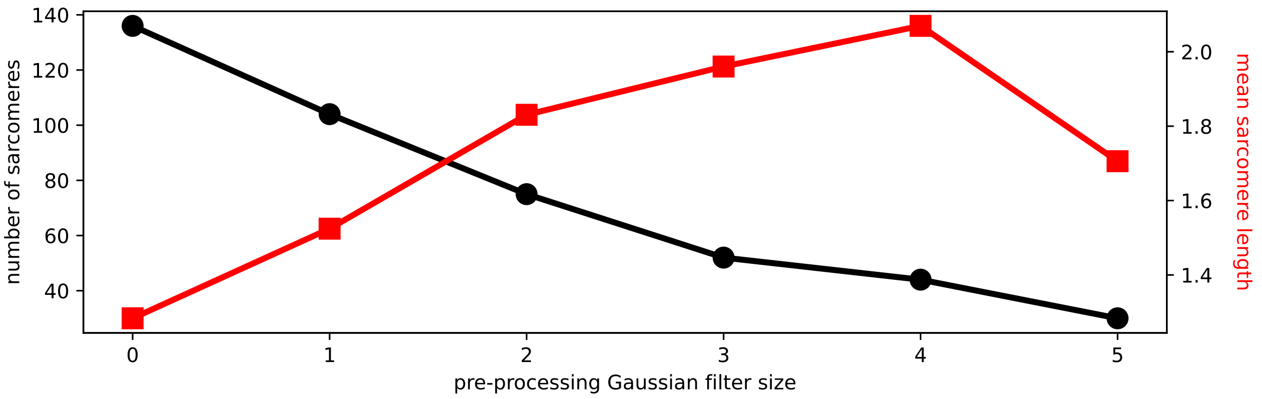

Distribution of mean sarcomere length segmented with Sarc-Graph for different Gaussian filter sizes:

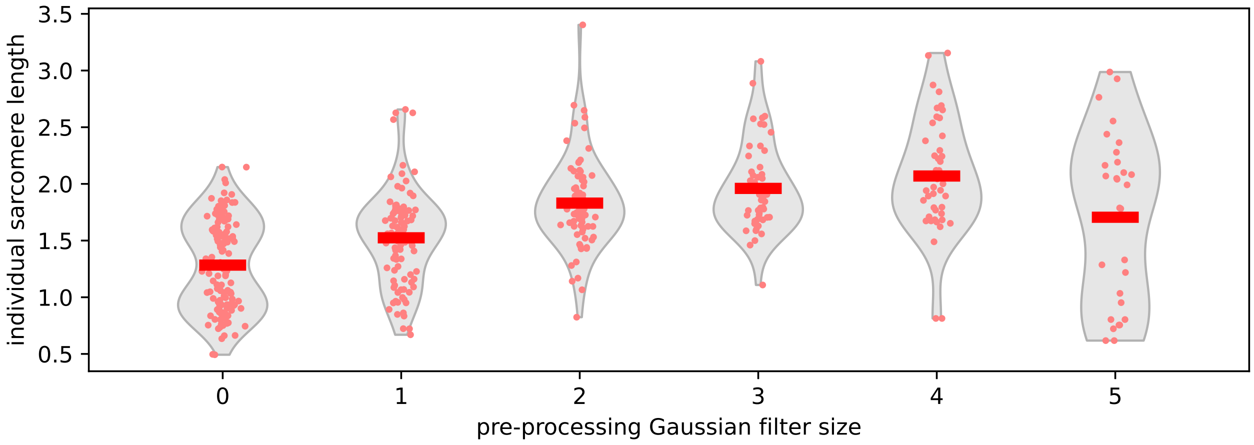

Note that for the Sarc-Graph default setting (no filter), the sarcomere length distribution in this image is *bimodal.* When we match the SOTA conditions and apply a Gaussian filter of size 4 to the image as a pre-processing step, the average length agrees well with the SOTA result ($\approx 2\,\mu m$). Also note that the measured sarcomere length may be influenced by the cell fixation process (see the section on immunocytochemistry in [1]).

To ensure that the observed bimodal distribution is not an artifact, we can visualize the bi-modal distribution of sarcomere lengths for images segmented without a pre-processing Gaussian filter (i.e., filter size = 0):

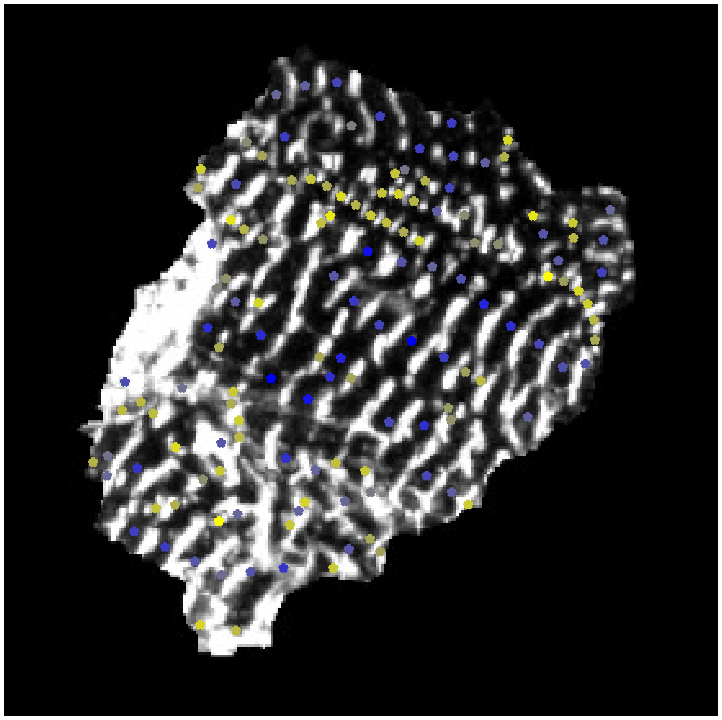

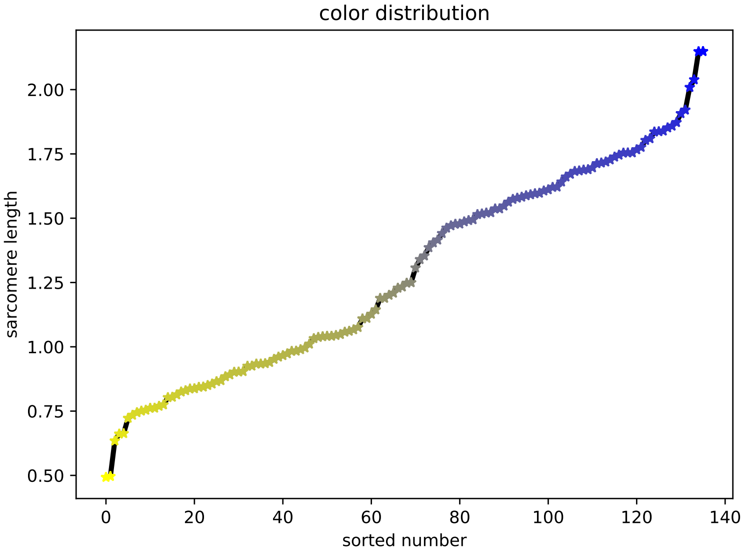

Briefly, we note that that some of the segmented sarcomeres shown above are likely spurious (e.g., bottom left corner). It is important to keep this in mind for formulating comparisons based on Sarc-Graph computed values.

Figure B. Sarcomere segmentation and analysis of the images shown in Fig 6 of Sutcliffe et al. 2018 [1]. In these images, sarcomeres segmented with Sarc-Graph are marked by a red star:

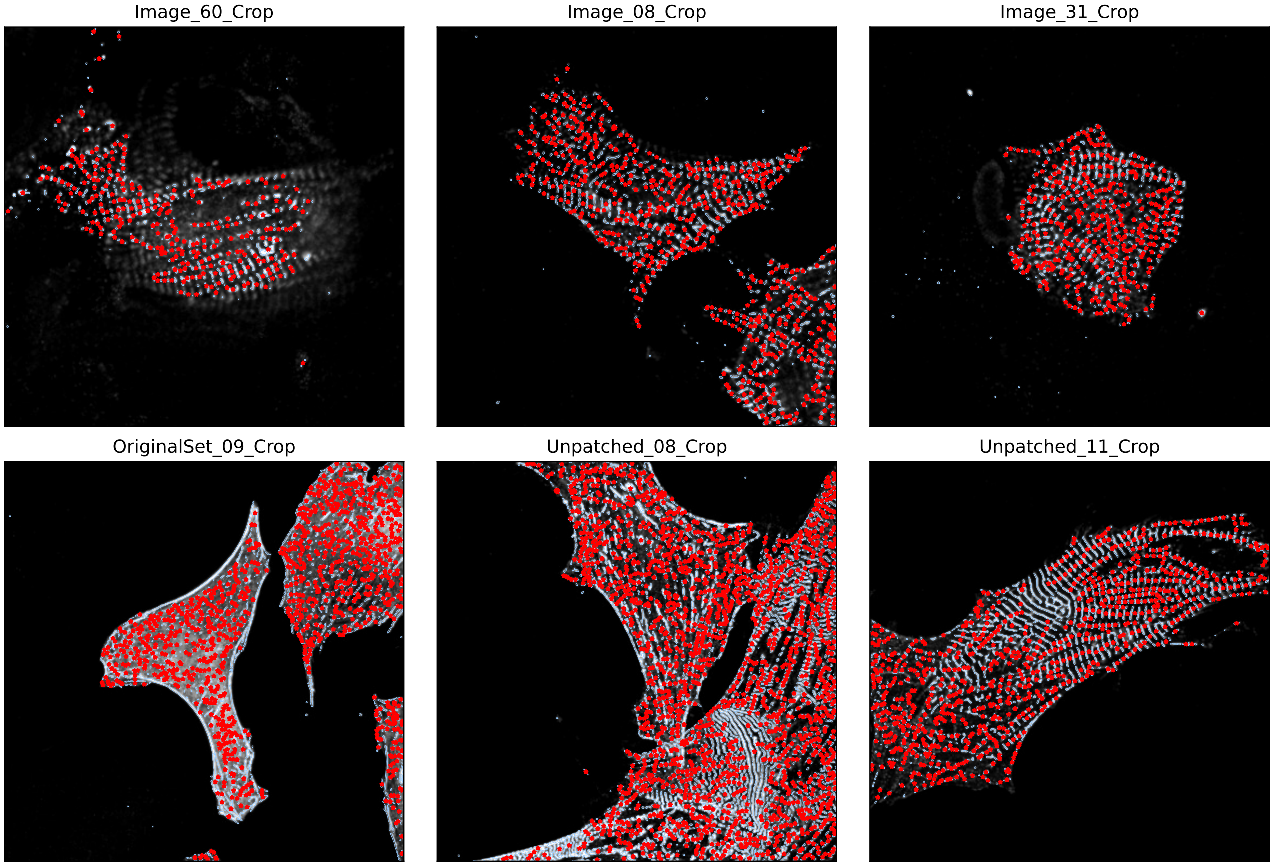

We note briefly that for all of image "09" and for part of image "08" discrete sarcomeres are not visible to the human eye (see [1] for the original figure). Though Sarc-Graph appears to be able to segment sarcomeres in these images, the code has not been validated for this case and thus this functionality should not be adopted without further investigation. *Even though the code detects sarcomeres, it is likely that the majority of them are spurious.* We also note that in the originally intended use, analyzing movies, only segmented objects that appear in multiple frames will be tracked and thus included in the final analysis. In the next two plots, note the qualitative differences in sarcomere length distribution. These differences could be used to specify which images are "valid" for analysis though this investigation is beyond the scope of this paper.

Number of sarcomeres and mean sarcomere length segmented with Sarc-Graph for the different images:

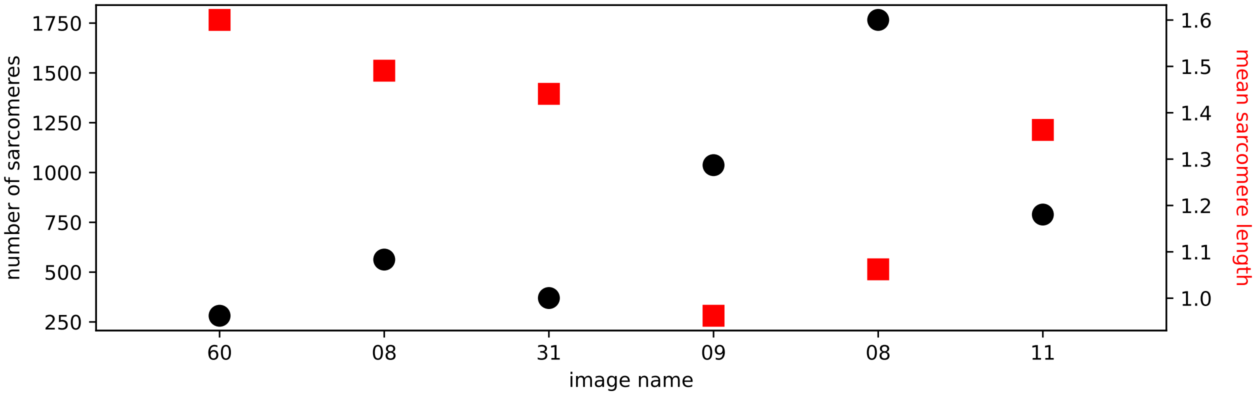

Distribution of mean sarcomere length segmented with Sarc-Graph for the different images:

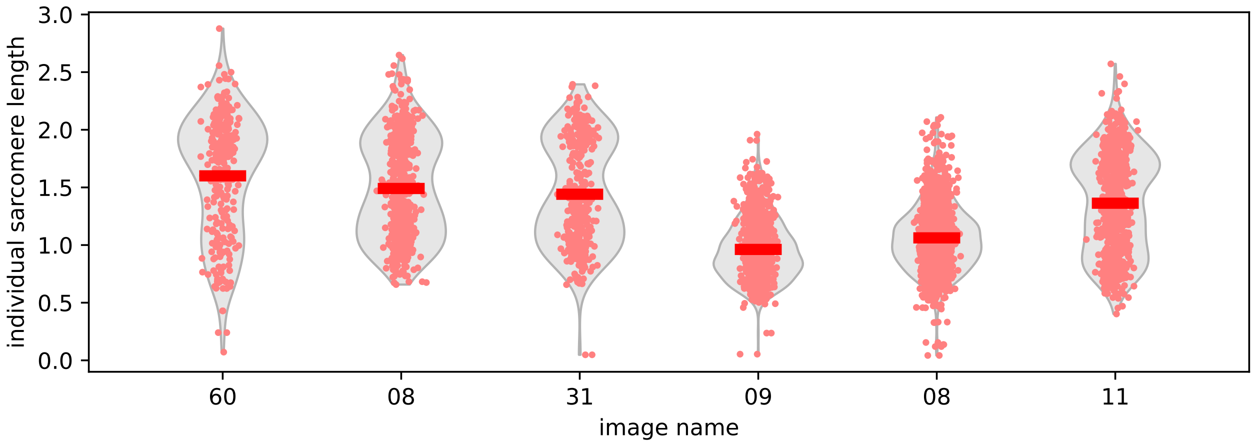

Direct comparison of average sarcomere length (μm) computed with SOTA and Sarc-Graph:

image number	SOTA [1]	Sarc-Graph (mean)	Sarc-Graph (median)
60	2.09	1.60	1.76
08	2.17	1.49	1.46
31	2.08	1.44	1.37
09	N/A	0.96	0.92
08	N/A	1.06	1.04
11	1.83	1.36	1.38

Again, we note that the bi-modal distribution of sarcomere lengths computed by Sarc-Graph indicates that a strategy other than mean sarcomere length comparison between images may be more appropriate.

References

1 Matthew D Sutcliffe, Philip M Tan, Antonio Fernandez-Perez, Young-Jae Nam, Nikhil V Munshi, and Jeffrey J Saucerman. High content analysis identifies unique morphological features of reprogrammed cardiomyocytes. *Scientific reports*, 8(1):1–11, 2018.
