# Supplementary material for: Sarc-Graph: Automated segmentation, tracking, and analysis of sarcomeres in hiPSC-derived cardiomyocytes
Source: PLoS Comput Biol. 2021 Oct 6;17(10):e1009443. doi: 10.1371/journal.pcbi.1009443 (PMC8523047; doi:10.1371/journal.pcbi.1009443)
Supplement: S2 Text — Evaluation of Sarc-Graph as a potential tool for creating features suitable for machine learning based classification. Fig A. Pasqualini et al. [6] dataset. Visualization of the un-processed dataset from Pasqualini et al. [6]. Fig B. Pasqualini et al. [6] dataset segmented. Visualization of the dataset from Pasqualini et al. [6] segmented with Sarc-Graph. Fig C. Pasqualini et al. [6] dataset analysis results. Visualization of the information obtained from the Sarc-Graph segmentation shown in Fig B in S2 Text. (PDF) [file pcbi.1009443.s002.pdf]

Sarc-Graph: Automated segmentation, tracking, and analysis of sarcomeres in hiPSC-derived cardiomyocytes
S2 Text · Pasqualini et al. 2015 [1] Comparison

Description of the comparison software:
In Pasqualini et al. 2015, "Structural Phenotyping of Stem Cell-Derived Cardiomyocytes," the authors define 11 metrics to capture stem cell-derived cardiomyocyte structural phenotype [1]. The ImageJ Scripts for computing these metrics are currently unavailable (see `http://diseasebiophysics.seas.harvard.edu/publications`, as of April 4th 2021, "DISEASE BIOPHYSICS GROUP CONFIDENTIAL, Unpublished Copyright (c) 2013, DISEASE BIOPHYSICS GROUP, All Rights Reserved."). The 11 metrics are: (1) sarcomere length; (2) total energy; (3) sarcomeric energy; (4) sarcomeric packing density; (5) orientational order parameter (OOP); (6) sarcomeric OOP (OOP1); (7) non sarcomeric OOP (OOP2); (8) z-disks relative presence; (9) weighted OOP; (10) coverage quality control; and (11) coherency quality control. A key finding from this paper is that using machine learning, it is possible to classify (score) images of stem cell-derived cardiomyoctyes based on these 11 metrics. Due to the unavailability of the code, we are not able to directly compare these metrics with our own. However, we are able to create a preliminary demonstration of the feasibility of using metrics defined by Sarc-Graph to create a machine learning model for structural phenotyping.

Description of the comparison data:
Here, we will use the comparison dataset provided with the paper [1] downloaded from `http://diseasebiophysics.seas.harvard.edu/publications` on April 4th 2021. This dataset contains 120 images of primary cardiomyocytes from neonate rats (rpCMs) matured for 6 hours, 123 images matured for 24 hours, and 123 images matured for 48 hours. These groups are referred to as "differentiated," "immature," and "mature" respectively. These images are of an $\alpha-$actinin stain. In this Appendix, we demonstrate that it is possible to use metrics derived from the Sarc-Graph segmentation step to classify these groups. Due to lack of data availability, we were unable to evaluate how this classification would extend to images of iPSC-CMs. We note briefly that Sarc-Graph is primarily for movies rather than still images. Critically, we anticipate that the functional metrics that Sarc-Graph defines from movies will also be useful for more advanced cell phenotyping via machine learning in the future.

Figure A. Here we show the raw images present in the dataset from Pasqualini et al. [1]. We have randomly (via setting a random seed in Python) selected three images from each group (6 hours, 24 hours, 48 hours) to show. Then, we convert each image (97 × 579 pixels) into a vector and use both principal component analysis (PCA) [1] and uniform manifold approximation and projection (UMAP) [1] to visualize the data. From these visualizations, it is apparent that the data is not readily separable by group based on the raw images alone using these standard techniques.

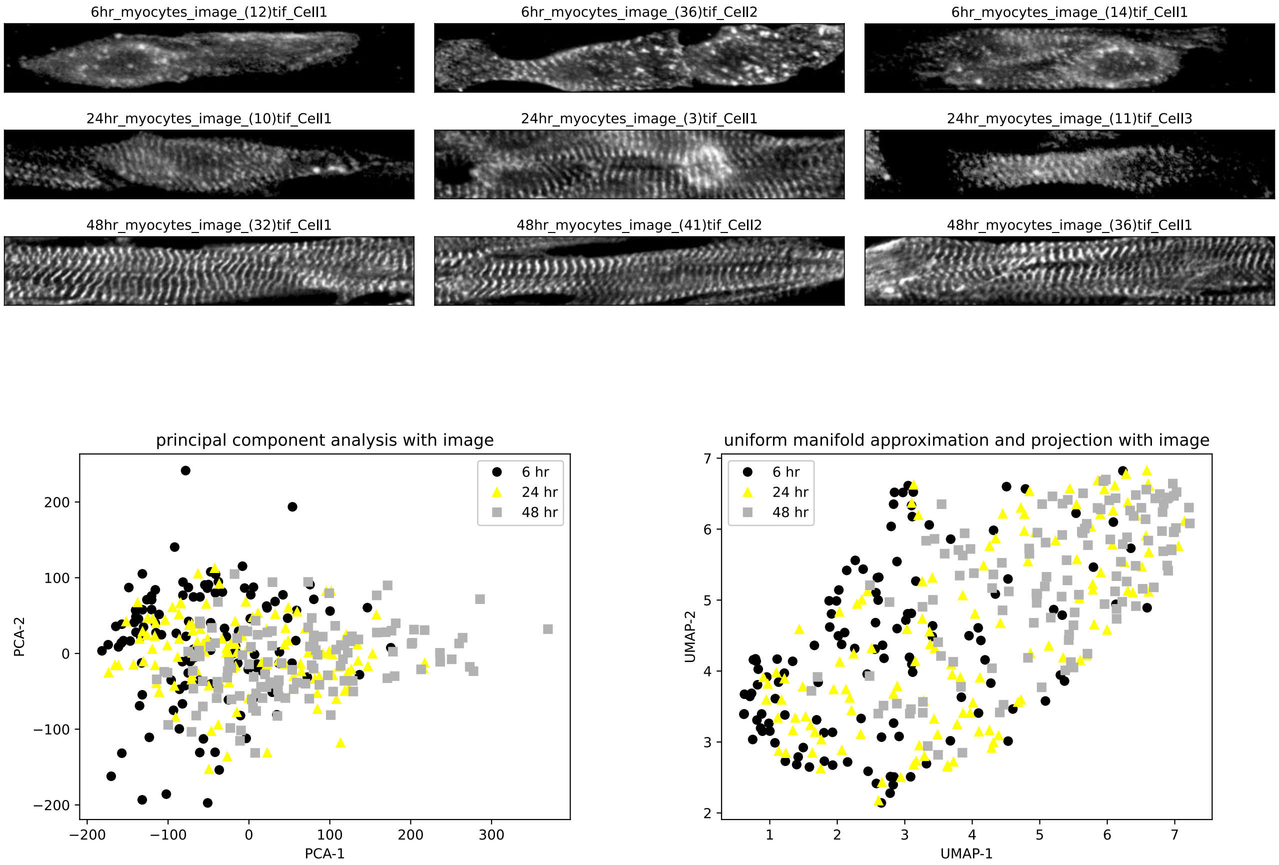

Figure B. Here we show these same images segmented with Sarc-Graph. The violin plots show the distribution of individual sarcomere length and width in pixels for these images. In these images, z-discs are outlined in light blue and segmented sarcomeres are indicated by a red dot. Of note, we set the parameter `gaussian_filter_size = 2`. And, for the "6 hr" group, there are many structures segmented as sarcomeres that may not be.

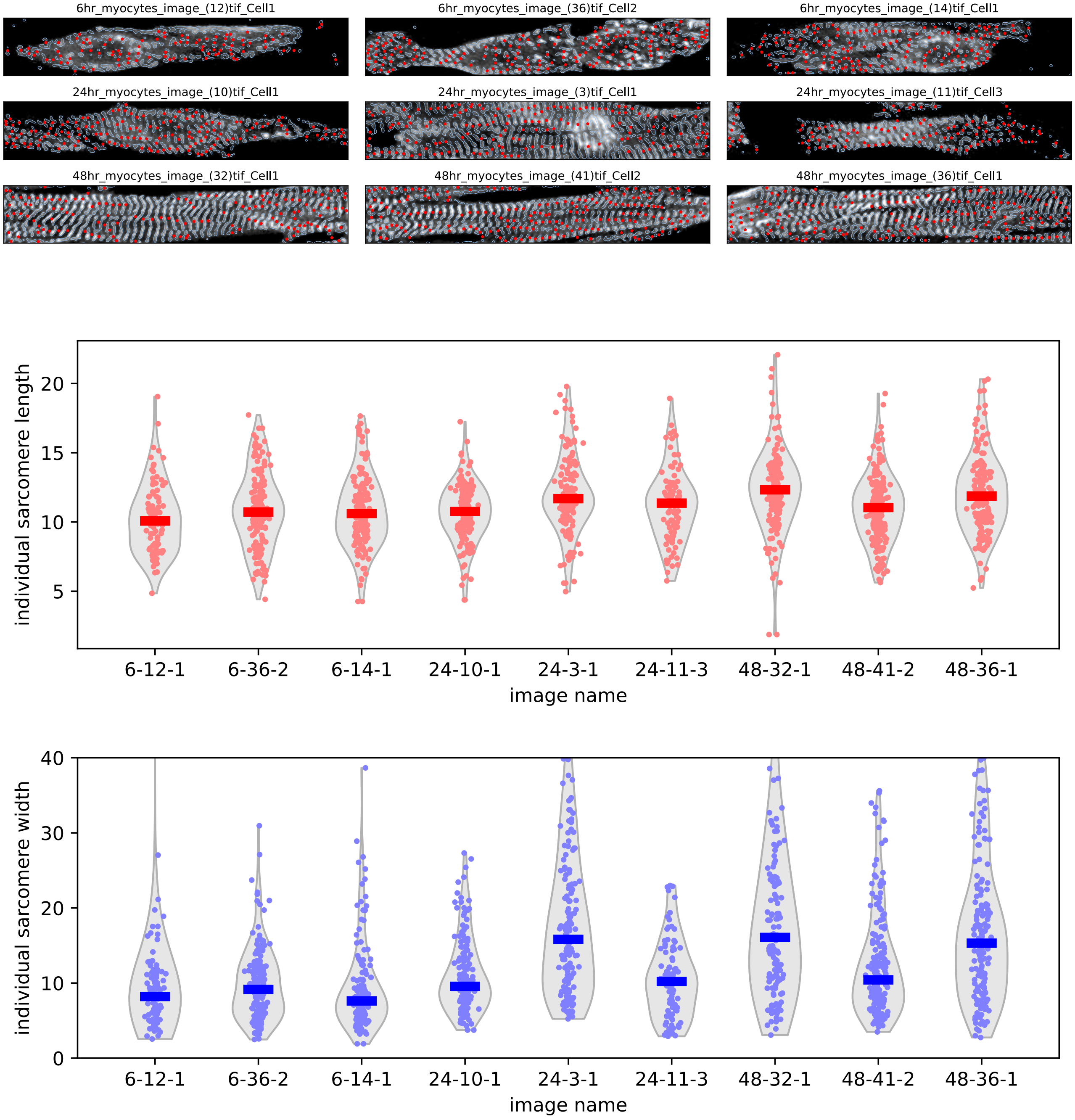

Figure C. Here we show the distribution of different features extracted by Sarc-Graph for all images in the dataset. In the histograms (mean sarcomere length, mean sarcomere width, number of segmented sarcomeres, OOP) subtle differences between groups are visually apparent. If we define each image by these four features, we can perform PCA and UMAP on the feature matrix. In comparison to Fig A, the resulting plots show that the groups are (qualitatively) much more clearly separated than before feature construction. Based on the results of this visualization, we anticipate that many standard machine learning methods could be used to classify these data based on Sarc-Graph features. Notably, metrics derived from movies rather than still images would likely give more functional insight.

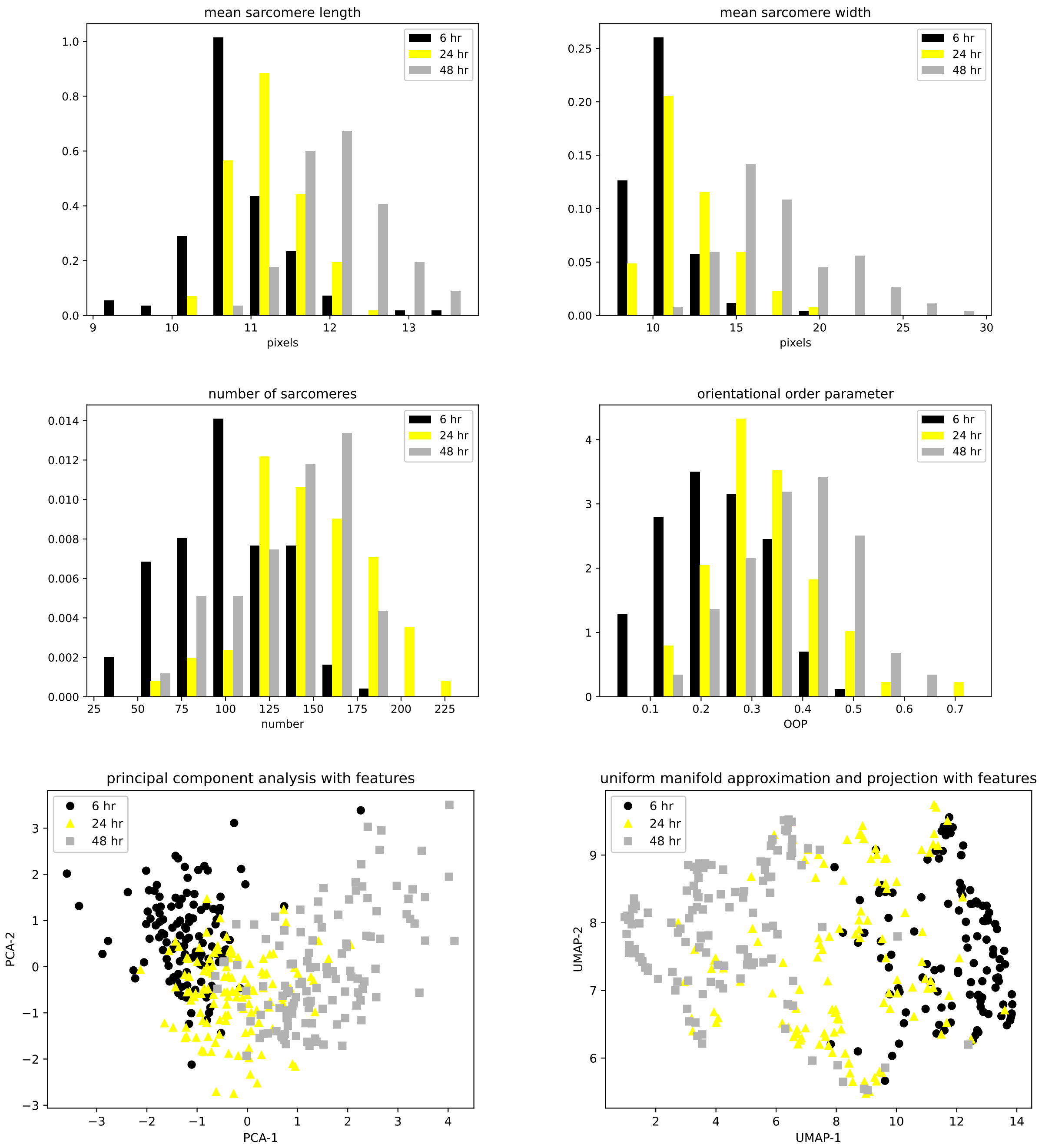

References

1 Francesco Silvio Pasqualini, Sean Paul Sheehy, Ashutosh Agarwal, Yvonne Aratyn-Schaus, and Kevin Kit Parker. Structural phenotyping of stem cell-derived cardiomyocytes. *Stem cell reports*, 4(3):340–347, 2015.
