# Supplementary material for: Sarc-Graph: Automated segmentation, tracking, and analysis of sarcomeres in hiPSC-derived cardiomyocytes
Source: PLoS Comput Biol. 2021 Oct 6;17(10):e1009443. doi: 10.1371/journal.pcbi.1009443 (PMC8523047; doi:10.1371/journal.pcbi.1009443)

Sarc-Graph: Automated segmentation, tracking, and analysis of sarcomeres in hiPSC-derived cardiomyocytes
S3 Text · Synthetic Data at Multiple Resolutions

Description of the data:
Here we perform a simple study with synthetic data to demonstrate the effect of image resolution on segmentation and tracking performance and the recovery of quantities of interest $\mathbf{F}_{\text{avg}}$ and OOP. The code to generate this synthetic data can be found on the Sarc-Graph GitHub page `https://github.com/elejeune11/Sarc-Graph`. Briefly, we generate three different example geometries, apply a ground truth homogeneous isotropic deformation to these geometries, and then *render* the geometry at multiple different resolutions (360×360 pixels, 180×180 pixels, 135×135 pixels, and 90×90 pixels). We chose these examples as (1) an unrealistically high resolution example (360×360) that leads to a much sharper z-disc than what would typically be seen in real experimental data, (2) a typical high resolution example (180×180), (3) a typical low resolution example (135×135), and (4) an example where Sarc-Graph does not function (90×90). Overall, we find that performance decreases as resolution decreases. However, we note that when Sarc-Graph is able to successfully segment and track sarcomeres, the resulting predicted QoI are a close match to the ground truth. In addition, we demonstrate that for these examples, Sarc-Graph error in predicting sarcomere angle distributions is related to the ability of Sarc-Graph to successfully segment z-discs. In general, Sarc-Graph is not overly sensitive to pixel error due to z-discs being small as long as the z-discs are sufficiently well defined to be segmented. Because z-disc size, shape, and fluorescent intensity will vary with respect to experimental conditions, it is difficult to define a specific rule of thumb for required resolution. However, these results indicate that if Sarc-Graph is able to segment z-discs the resulting metrics will not be overly sensitive to the relative resolution. Looking forward, future users concerned about specific types of error can conduct additional specific studies with our flexible synthetic data generation pipeline.

Figure A. Performance of Sarc-Graph in segmentation and tracking with respect to image resolution. The number in the box shows the number of sarcomeres successfully segmented and tracked out of 50 for each movie. The lower two rows show multiple still frames from the synthetic data movies. In the low resolution images, segmentation is more difficult and tracking is more likely to fail due to the inconsistencies in segmentation.

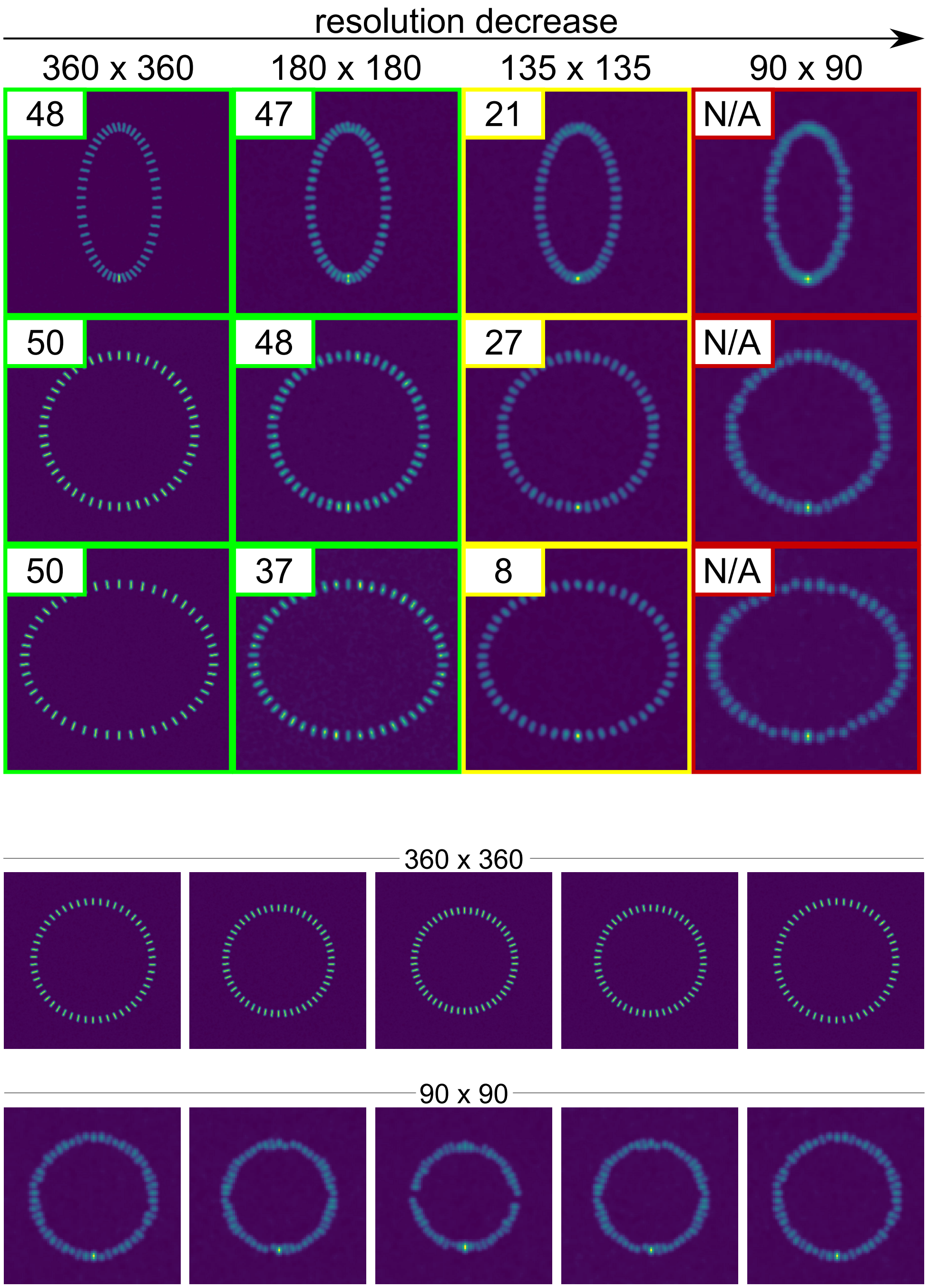

Figure B. Comparison of $\mathbf{F}_{\text{avg}}$ computed with Sarc-Graph to the ground truth at different rendering resolutions. Note that the mean squared error (MSE) stays low until Sarc-Graph fails at 90x90 pixels.

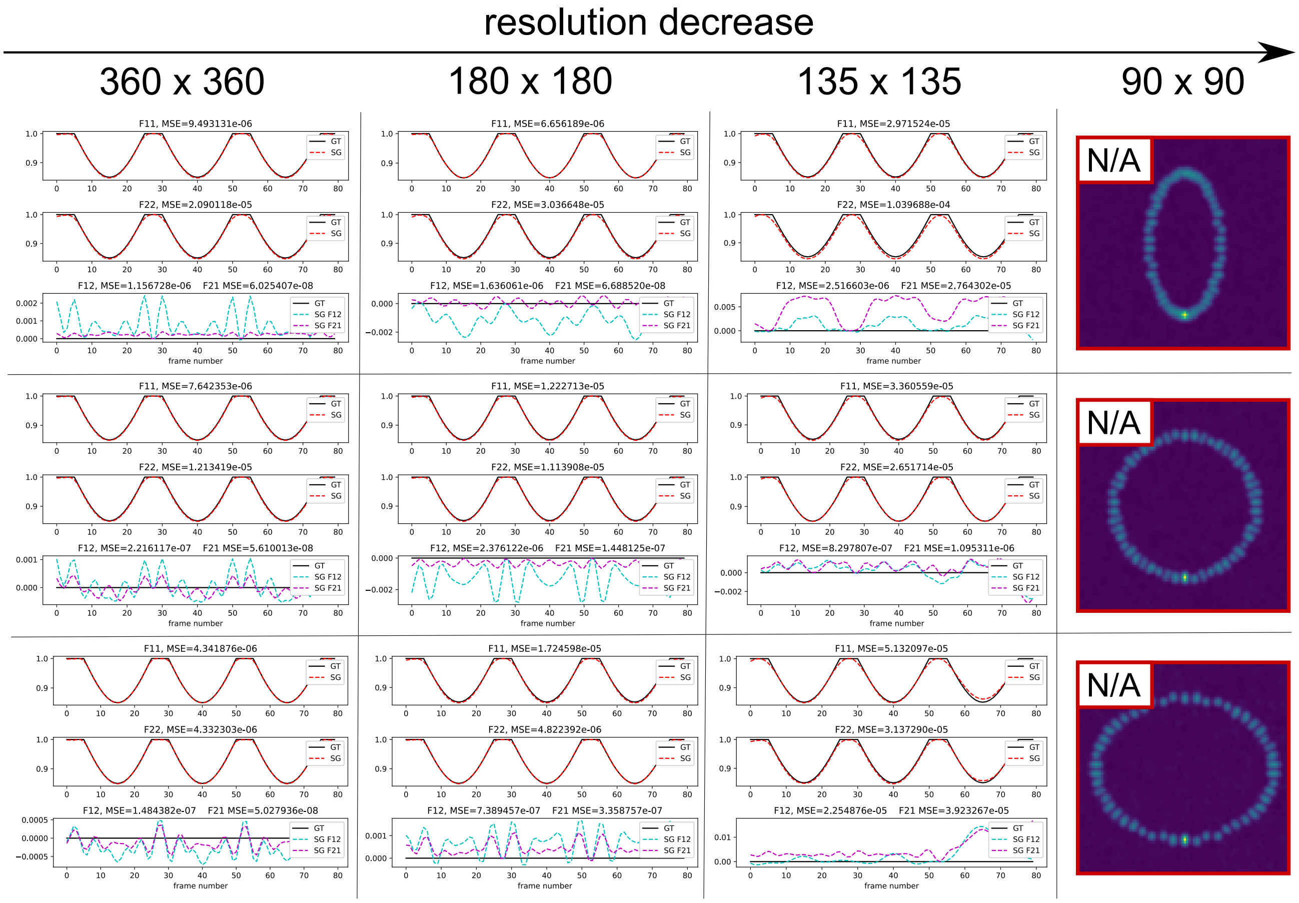

Figure C. Comparison of OOP computed with Sarc-Graph to the ground truth at different rendering resolutions. Note that the results are consistent until Sarc-Graph fails at 90x90 pixels.

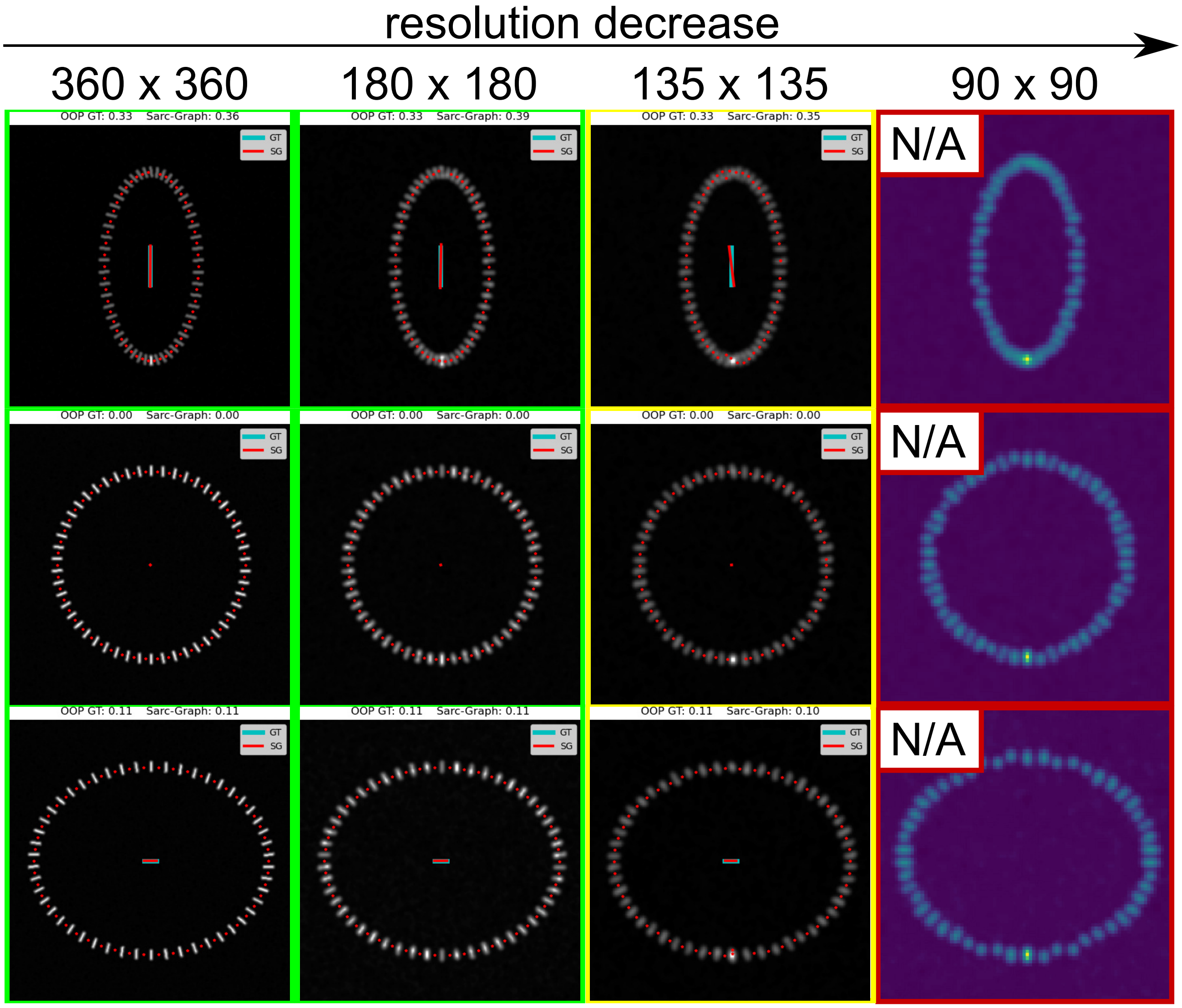

Figure D. Comparison of sarcomere angle computed with Sarc-Graph to the ground truth at different rendering resolutions. Note that recovery of the ground truth degrades as resolution decreases.

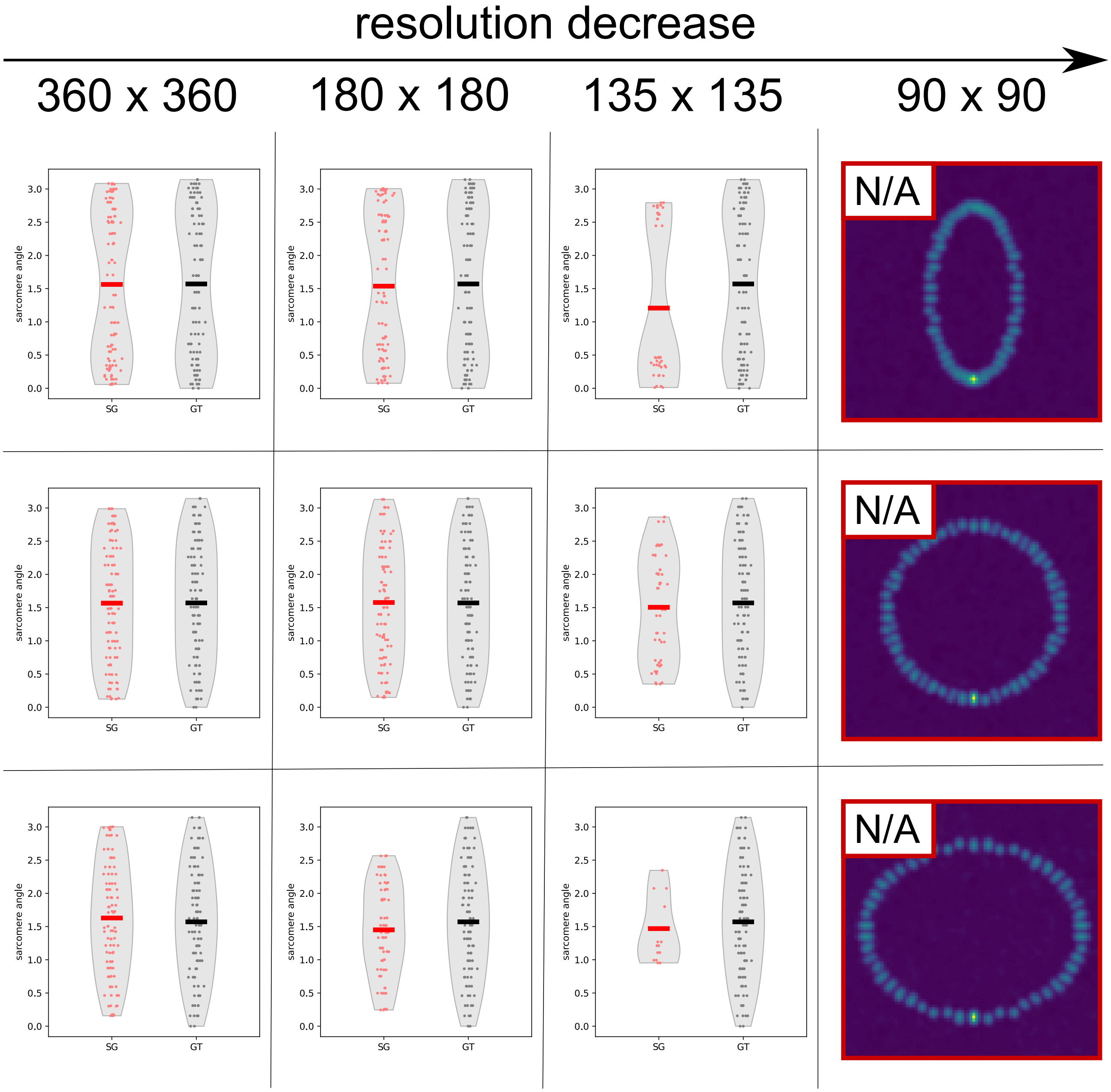

Supplement: S3 Text — Presentation and analysis of more synthetic data at multiple image resolutions (this is similar to Fig 5). Fig A. Demonstration of varied image resolution. Performance of Sarc-Graph in segmentation and tracking with respect to image resolution. Fig B. Favg analysis at multiple image resolutions. Comparison of Favg computed with Sarc-Graph to the ground truth at different rendering resolutions. Fig C. OOP analysis at multiple image resolutions. Comparison of OOP computed with Sarc-Graph to the ground truth at different rendering resolutions. Fig D. Angle analysis at multiple image resolutions. Comparison of sarcomere angle computed with Sarc-Graph to the ground truth at different rendering resolutions. (PDF) [file pcbi.1009443.s003.pdf]
