# Supplementary material for: Sarc-Graph: Automated segmentation, tracking, and analysis of sarcomeres in hiPSC-derived cardiomyocytes
Source: PLoS Comput Biol. 2021 Oct 6;17(10):e1009443. doi: 10.1371/journal.pcbi.1009443 (PMC8523047; doi:10.1371/journal.pcbi.1009443)
Supplement: S4 Text — Direct comparison to an alternative method for automated segmentation and tracking of hiPSC-CMs. Fig A. SarcTrack [10] comparison 1. Synthetic data and comparison to SarcTrack [10], example “411.” Fig B. SarcTrack [10] comparison 2. Synthetic data and comparison to SarcTrack [10], example “412.” Fig C. SarcTrack [10] comparison 3. Synthetic data and comparison to SarcTrack [10], example “421.” Fig D. SarcTrack [10] comparison 4. Synthetic data and comparison to SarcTrack [10], example “422.” Fig E. SarcTrack [10] comparison 5. Synthetic data and comparison to SarcTrack [10], example “1011.” Fig F. SarcTrack [10] comparison 6. Synthetic data and comparison to SarcTrack [10], example “1012.” Fig G. SarcTrack [10] comparison 7. Synthetic data and comparison to SarcTrack [10], example “1021.” Fig H. SarcTrack [10] comparison 8. Synthetic data and comparison to SarcTrack [10], example “1022.”. (PDF) [file pcbi.1009443.s004.pdf]

Sarc-Graph: Automated segmentation, tracking, and analysis of sarcomeres in hiPSC-derived cardiomyocytes
S4 Text · Toepfer et al. 2019 [1] Comparison

Description of the comparison software:
SarcTrack is a software for segmenting, tracking, and analyzing sarcomeres in movies of fluorescently labeled hiPSC-CMs [1]. To our knowledge, it is the open source software most related to Sarc-Graph. To perform the enclosed analysis, we downloaded SarcTrack from `https://github.com/HMS-IDAC/SarcTrack` on March 23rd 2021 and ran it using MATLAB R2019a. For reference, key differences between Sarc-Graph and SarcTrack are as follows:

- Sarc-Graph is Python based whereas SarcTrack is MATLAB based.
- Sarc-Graph and SarcTrack use fundamentally different methodological approaches for both segmentation and tracking.
- SarcTrack assumes that sarcomere contraction will follow a sawtooth curve whereas Sarc-Graph makes no assumption about the shape of the sarcomere time series.
- SarcTrack requires a manual parameter tuning step, whereas Sarc-Graph typically does not. In the reported data, we use the SarcTrack parameter settings provided with the example code for the synthetic dataset.
- Sarc-Graph provides multiple analysis and visualization tools beyond the scope of SarcTrack.

Description of the comparison data:
The comparison data is synthetic data originally generated for validating SarcTrack. The code to generate the synthetic data was downloaded from `https://github.com/HMS-IDAC/SarcTrack` on March 23rd 2021 and run using MATLAB R2019a. At the time of publication, these movies are also available from `https://www.dropbox.com/s/k1p65tnfeixp9q0/SarcTrackSampleVideos.zip?dl=0`. We analyze all eight of the provided synthetic data examples with both SarcTrack and Sarc-Graph. The eight movies are titled: "411," "412," "421," "422," "1011," "1012," "1021," and "1022." The number "4" corresponds to ground truth contraction of 0.4 pixels, the number "10" corresponds to ground truth contraction of "1.0" pixels, and the second two digits refer to the ratio between contraction and relaxation in the synthetic sawtooth contraction function (i.e., "1 : 2"). In all movies, Sarc-Graph is able to successfully track all 40 synthetic sarcomeres as discrete entities. Given the recommended parameter set (`ds = 9:0.2:11`, `stretch = 1`, `scale = 1.5`, `nangs = 8`, `hopsize = 7`, `halfwindowsize = 2`), SarcTrack picks up and tracks ≈ 80 points per movie. Detailed results for all movies provided are as follows. In general, the performance of the two softwares is similar but not identical. For the larger levels of sarcomere contraction (Fig E-H) Sarc-Graph captures the ground truth change in sarcomere length with a lower mean square error. We note that because these synthetic sarcomeres deform following near perfect sawtooth functions, these data represent a best case scenario for SarcTrack.

Figure A. Synthetic data and comparison to SarcTrack, example "411."

Segment, track, and analyze individual sarcomere time series with Sarc-Graph:

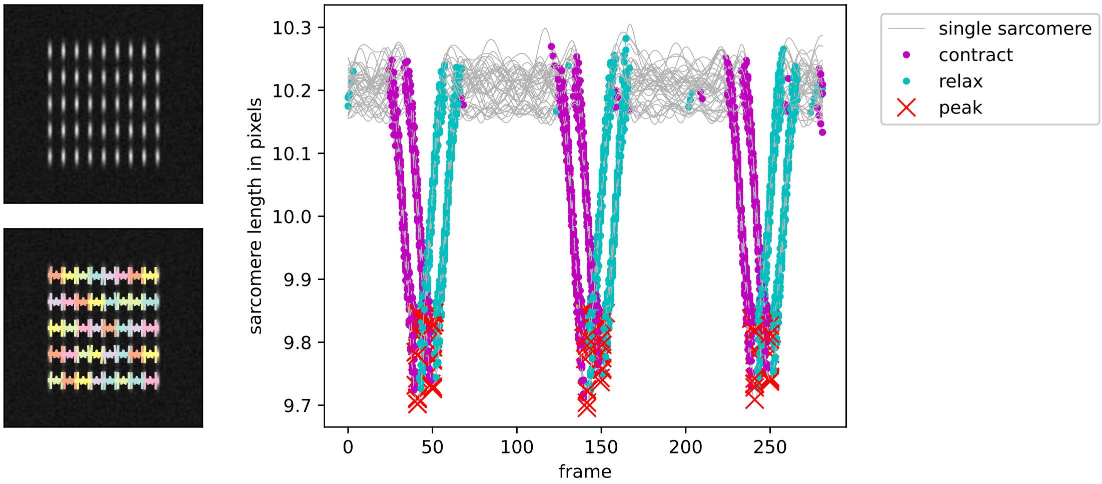

Compare Sarc-Graph (SG) results to the mean registered ground truth and SarcTrack (ST):

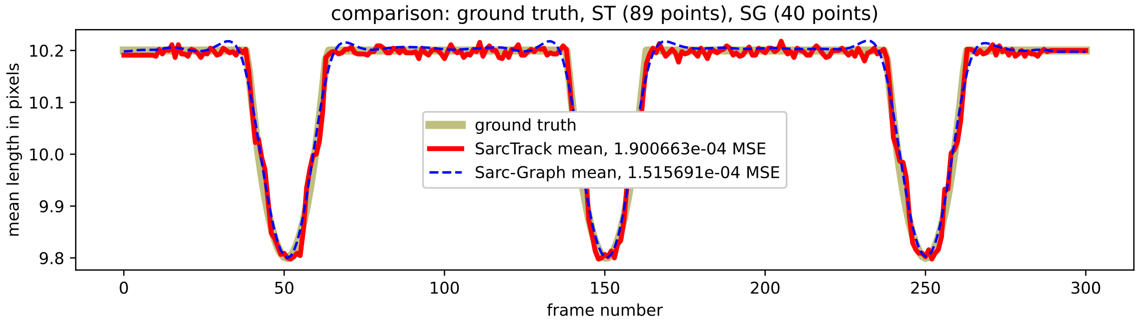

Compare measured QoI for SarcTrack and Sarc-Graph:

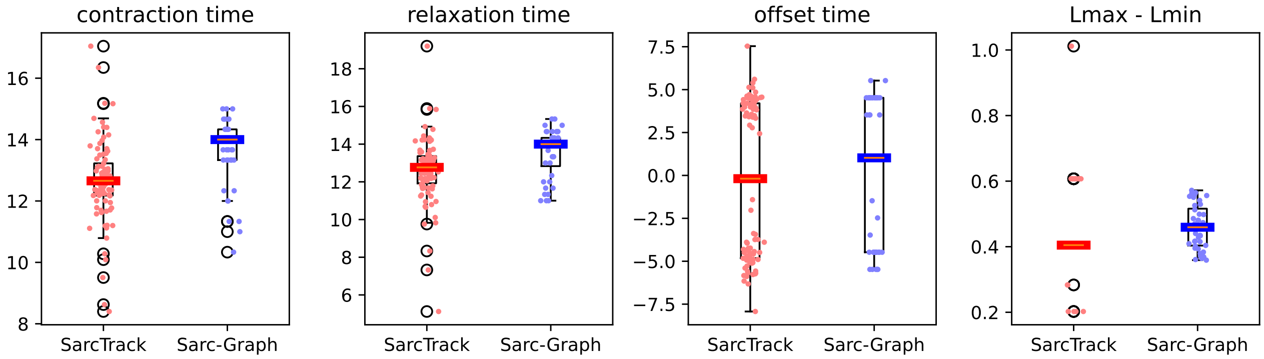

Figure B. Synthetic data and comparison to SarcTrack, example "412."

Segment, track, and analyze individual sarcomere time series with Sarc-Graph:

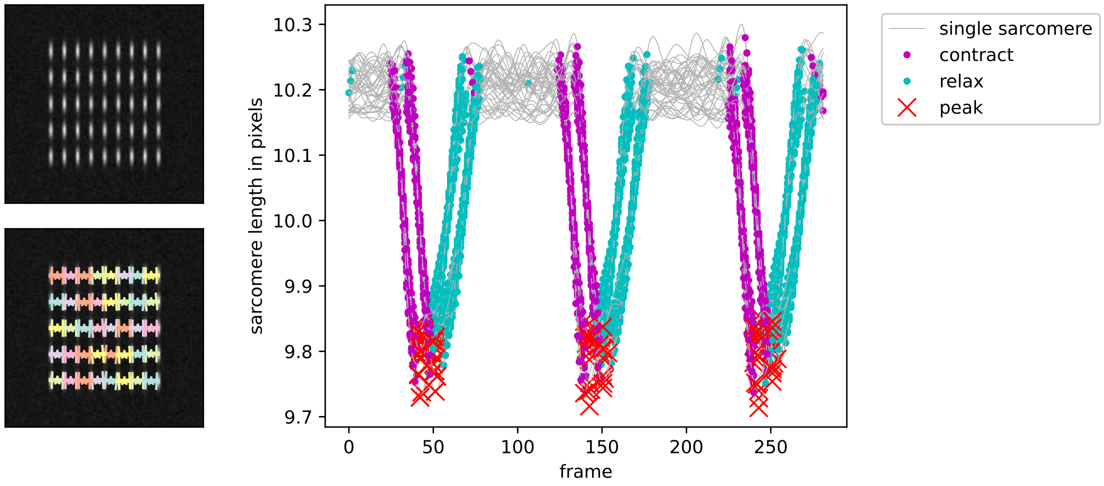

Compare Sarc-Graph (SG) results to the mean registered ground truth and SarcTrack (ST):

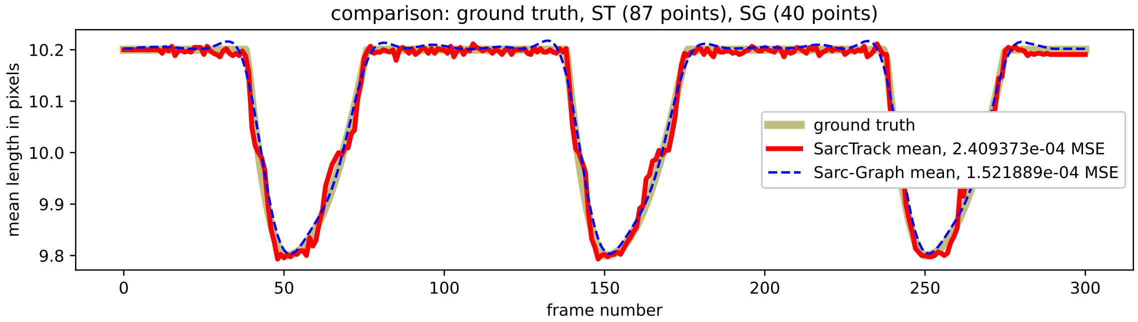

Compare measured QoI for SarcTrack and Sarc-Graph:

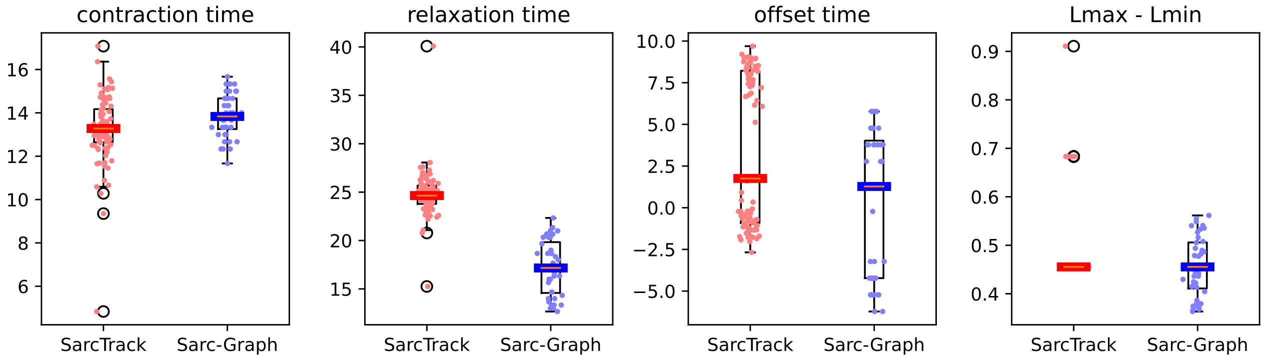

Figure C. Synthetic data and comparison to SarcTrack, example "421."

Segment, track, and analyze individual sarcomere time series with Sarc-Graph:

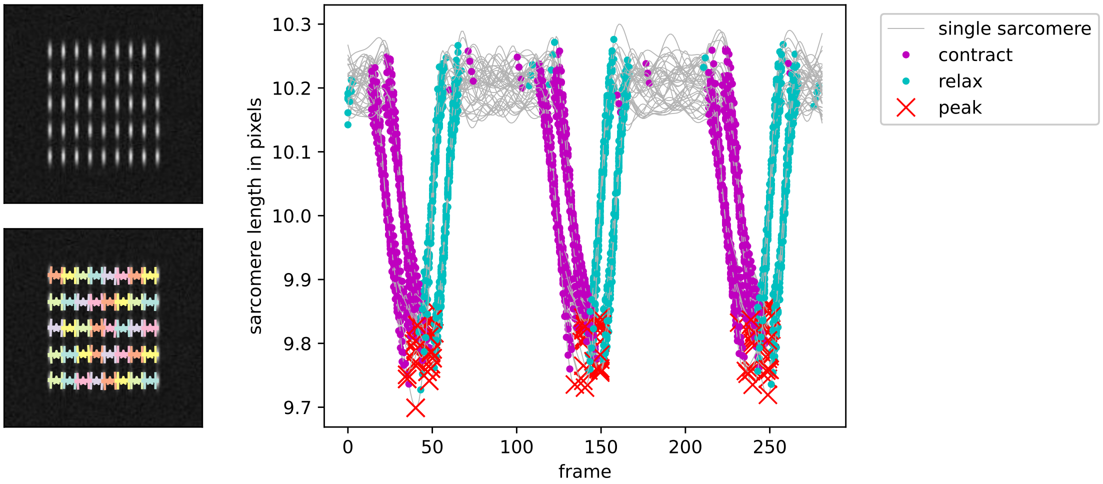

Compare Sarc-Graph (SG) results to the mean registered ground truth and SarcTrack (ST):

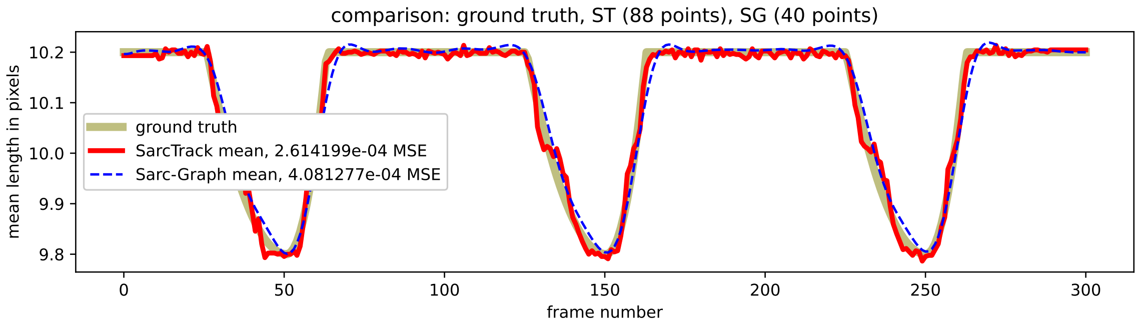

Compare measured QoI for SarcTrack and Sarc-Graph:

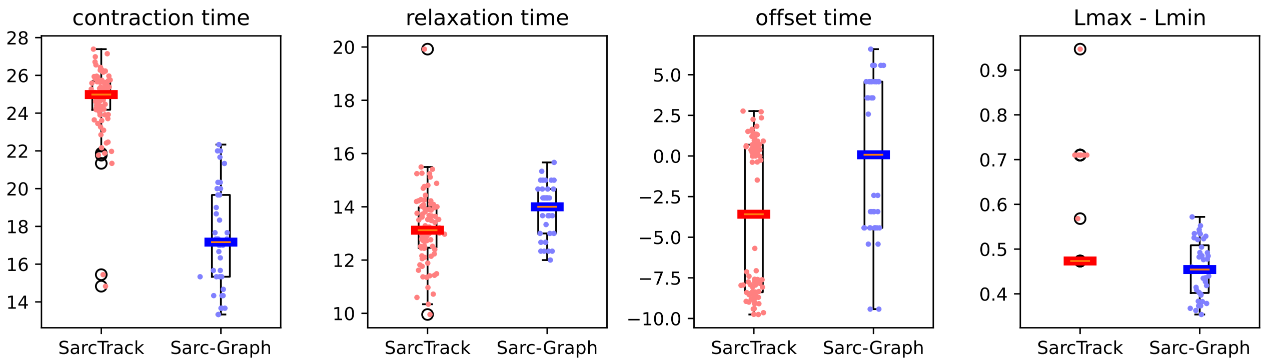

Figure D. Synthetic data and comparison to SarcTrack, example "422."

Segment, track, and analyze individual sarcomere time series with Sarc-Graph:

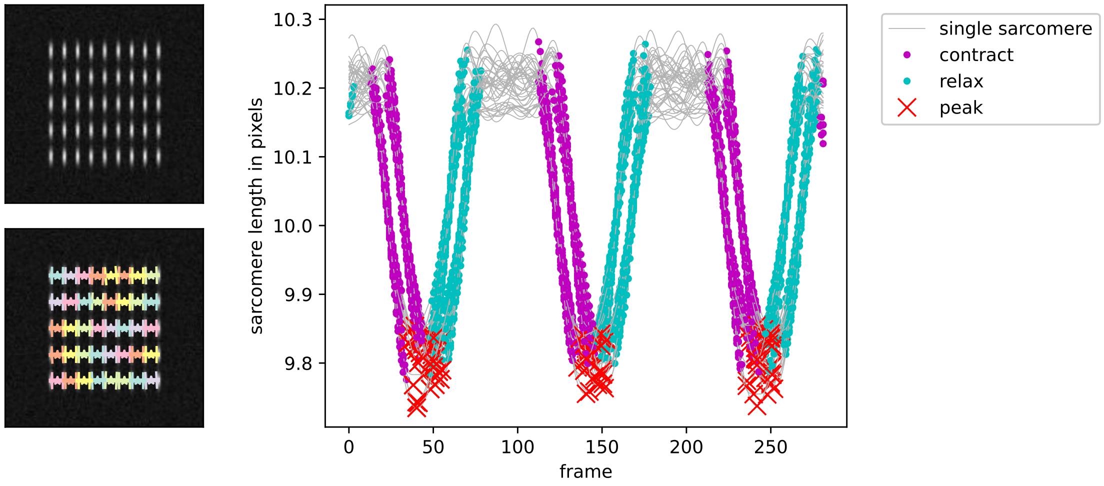

Compare Sarc-Graph (SG) results to the mean registered ground truth and SarcTrack (ST):

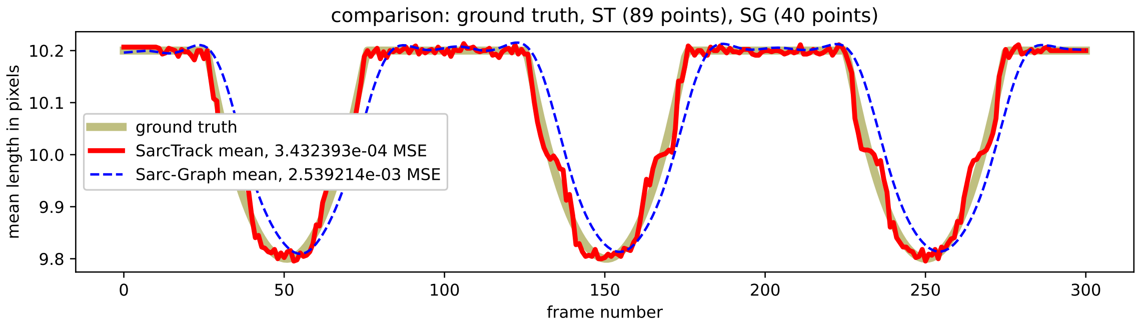

Compare measured QoI for SarcTrack and Sarc-Graph:

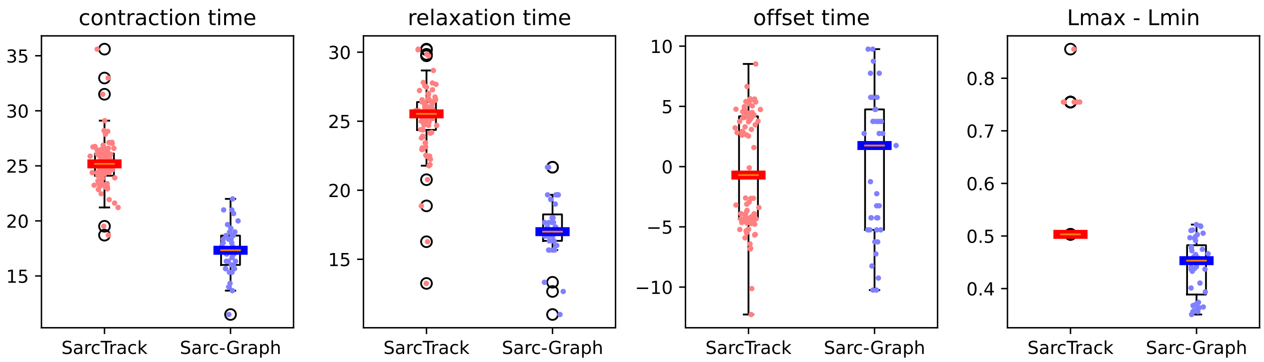

Figure E. Synthetic data and comparison to SarcTrack, example "1011."
Segment, track, and analyze individual sarcomere time series with Sarc-Graph:

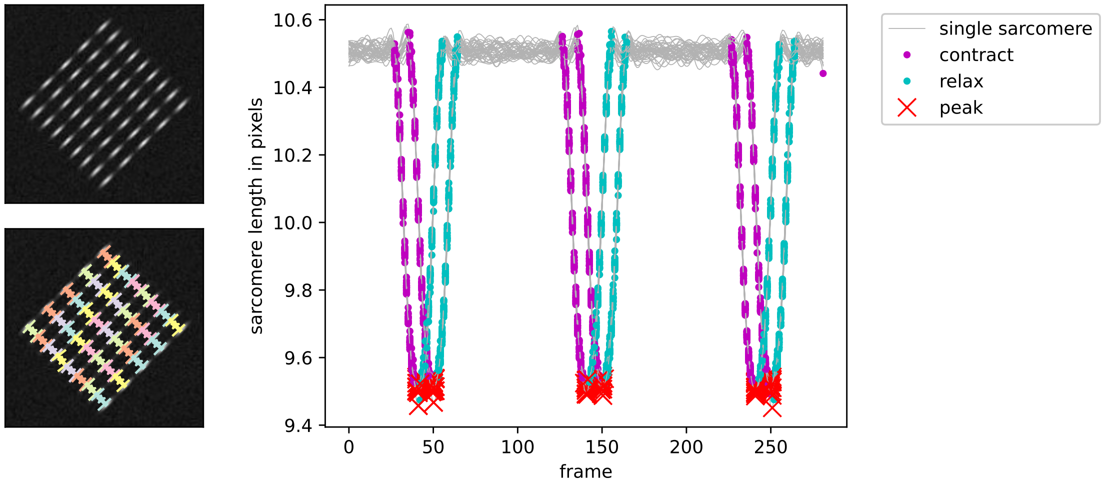

Compare Sarc-Graph (SG) results to the mean registered ground truth and SarcTrack (ST):

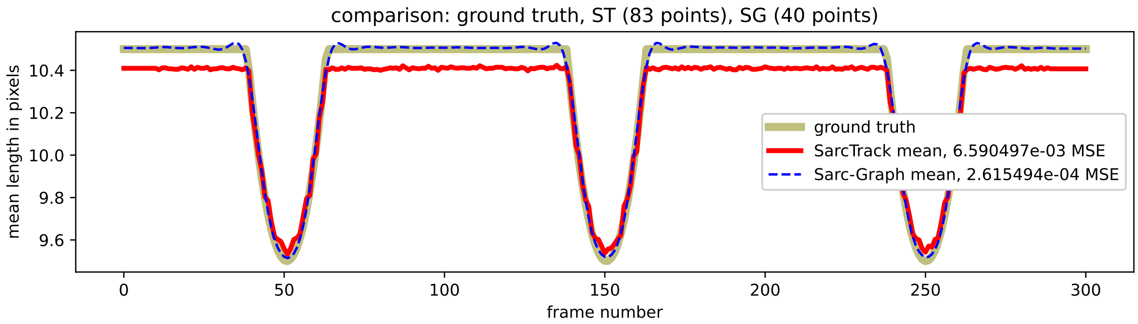

Compare measured QoI for SarcTrack and Sarc-Graph:

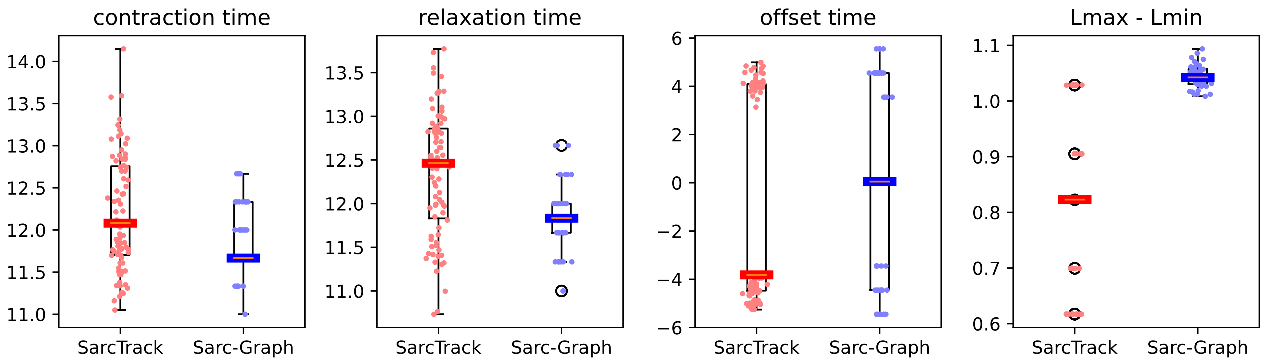

Figure F. Synthetic data and comparison to SarcTrack, example “1012.”

Segment, track, and analyze individual sarcomere time series with Sarc-Graph:

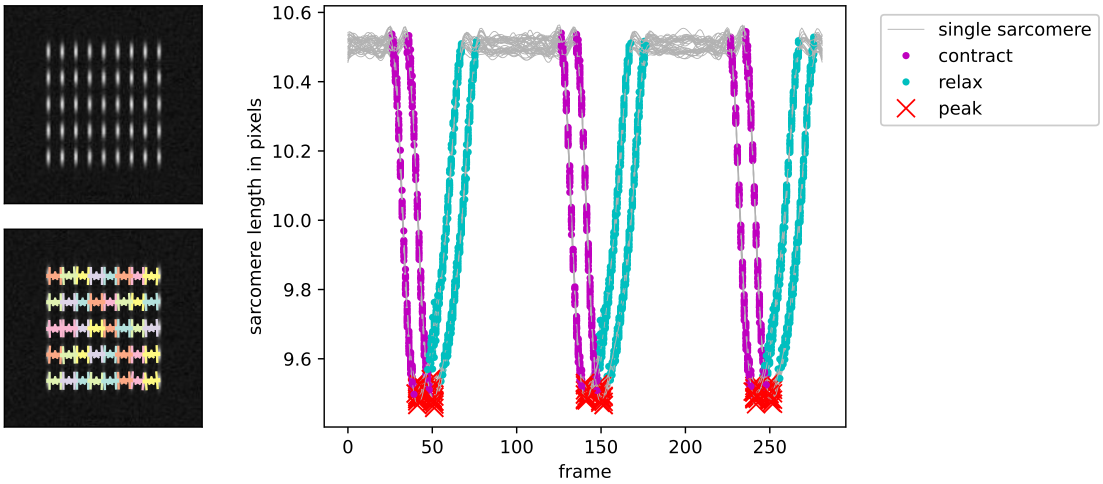

Compare Sarc-Graph (SG) results to the mean registered ground truth and SarcTrack (ST):

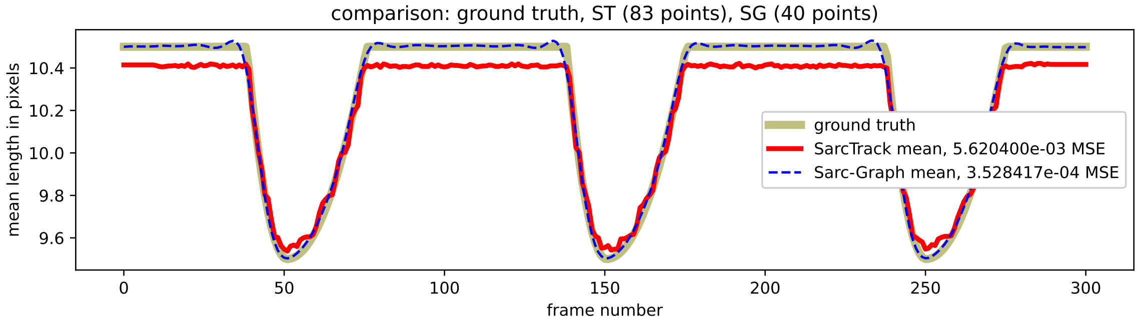

Compare measured QoI for SarcTrack and Sarc-Graph:

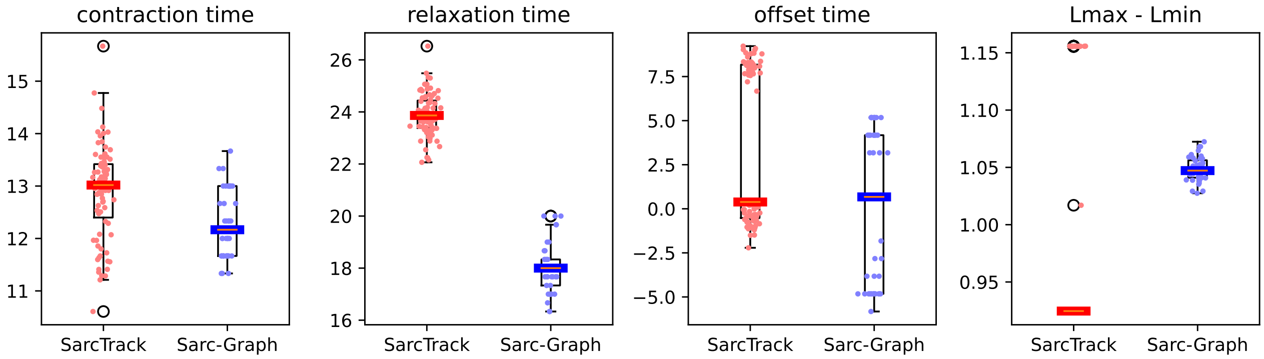

Figure G. Synthetic data and comparison to SarcTrack, example "1021."

Segment, track, and analyze individual sarcomere time series with Sarc-Graph:

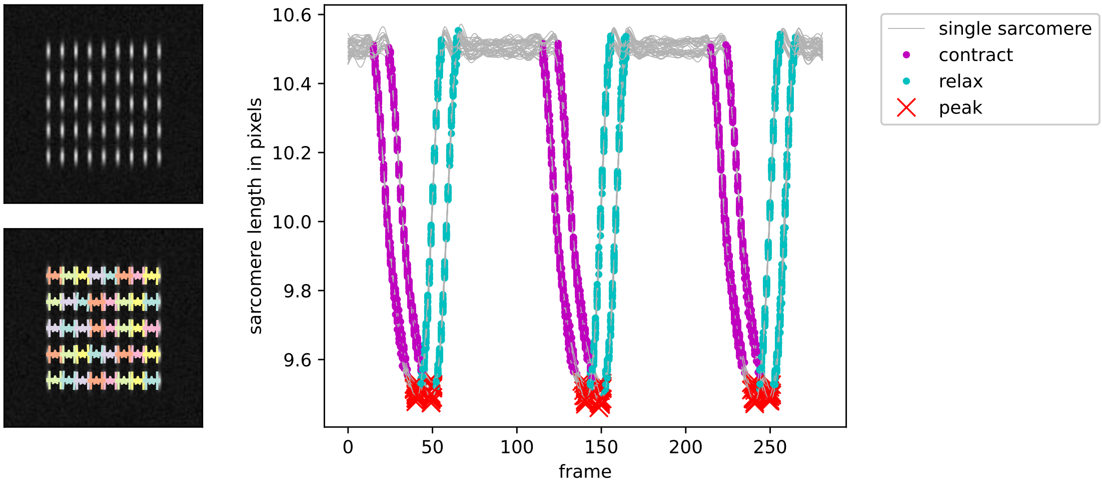

Compare Sarc-Graph (SG) results to the mean registered ground truth and SarcTrack (ST):

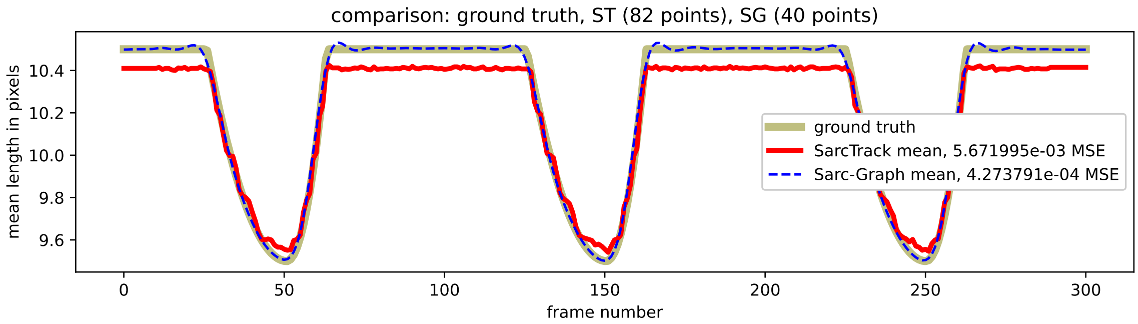

Compare measured QoI for SarcTrack and Sarc-Graph:

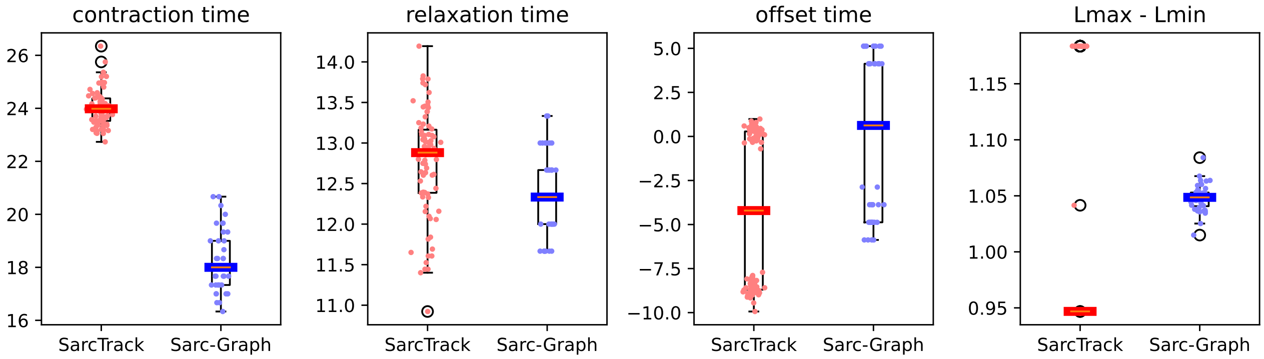

Figure H. Synthetic data and comparison to SarcTrack, example “1022.”
Segment, track, and analyze individual sarcomere time series with Sarc-Graph:

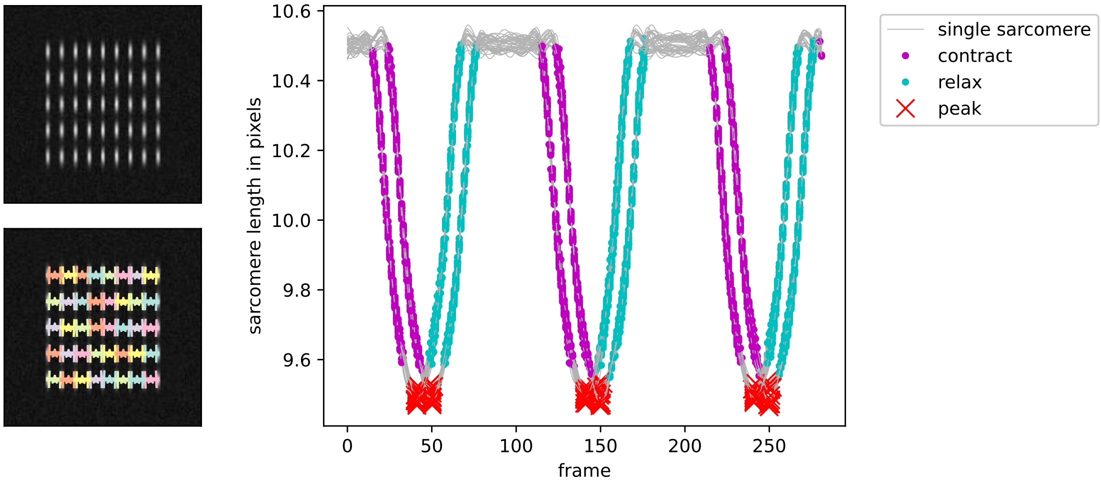

Compare Sarc-Graph (SG) results to the mean registered ground truth and SarcTrack (ST):

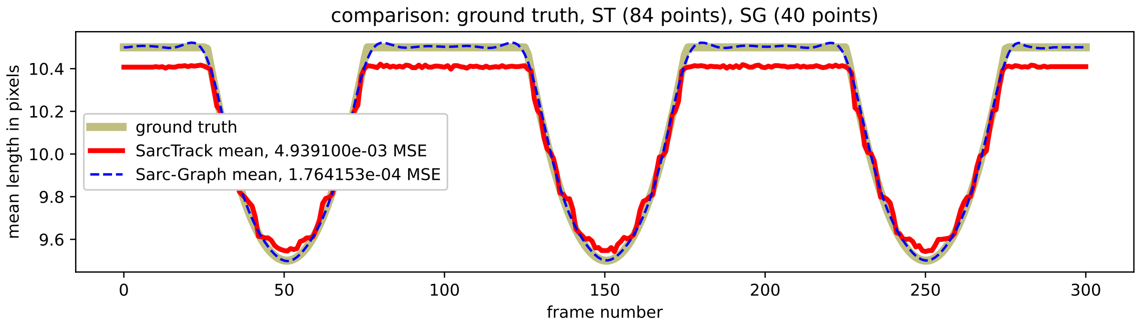

Compare measured QoI for SarcTrack and Sarc-Graph:

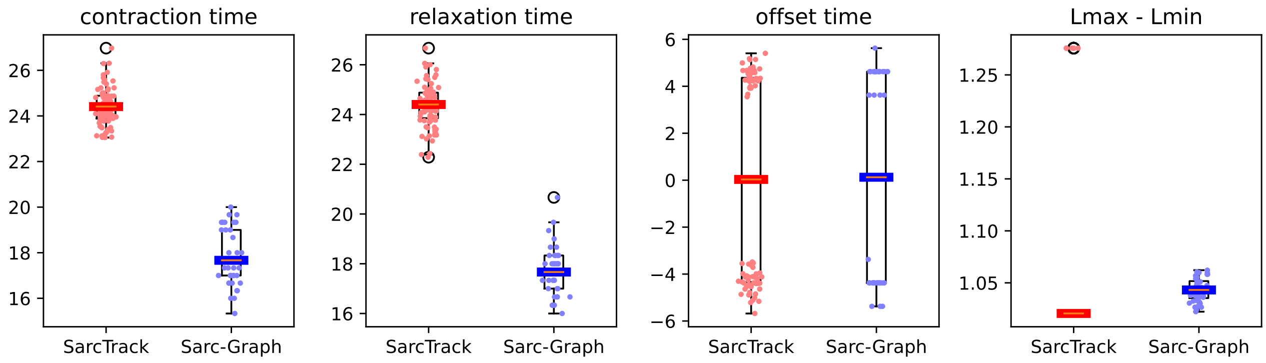

References

1 Christopher N Toepfer, Arun Sharma, Marcelo Cicconet, Amanda C Garfinkel, Michael Mücke, Meraj Neyazi, Jon AL Willcox, Radhika Agarwal, Manuel Schmid, Jyoti Rao, et al. Sarctrack: an adaptable software tool for efficient large-scale analysis of sarcomere function in hipsc-cardiomyocytes. *Circulation research*, 124(8):1172–1183, 2019.
