# Supplementary material for: Sarc-Graph: Automated segmentation, tracking, and analysis of sarcomeres in hiPSC-derived cardiomyocytes
Source: PLoS Comput Biol. 2021 Oct 6;17(10):e1009443. doi: 10.1371/journal.pcbi.1009443 (PMC8523047; doi:10.1371/journal.pcbi.1009443)
Supplement: Attachment — Submitted filename: rebuttal.pdf [file pcbi.1009443.s010.pdf]

PLOS Computational Biology (PCOMPBIOL-D-21-00225) - [EMID:6864f4224b90f156]
Response to Reviewers
Sarc-Graph: Automated segmentation, tracking, and analysis of sarcomeres in hiPSC-derived cardiomyocytes

---

We would like to begin by thanking the Editor and the Reviewers for taking the time to review our manuscript. The changes are pointed out in this rebuttal letter and are highlighted in red font in the attached manuscript. Critically, we have added four Appendix Text documents to the revised version of the manuscript in direct response to reviewer concerns:

- ***S1 Text** Sutcliffe et al. 2018 comparison.*
- ***S2 Text** Pasqualini et al. 2015 comparison.*
- ***S3 Text** Additional investigation of image resolution.*
- ***S4 Text** Toepher et al. 2019 comparison.*

Thank you for considering our revised submission.

Referee 1 Comments & Response to Reviewer

This manuscript describes a method to automatically track sarcomeres in hiPSC derived cardiomyocytes. This is an important problem that has been the subject of multiple publications lately as it is imperative that stem-cell derived cardiomyocytes are compared without bias among test populations. The method uses segmentation to track sarcomeric architecture through time and the outputs include metrics that quantify a measure of contractility, sarcomere shortening, and others. While this article addresses an important issue in the field, there are some major issues that need to be addressed prior to proper review:
Major:

1. The introduction reads like a discussion section in that it presents the results of the paper instead of the background. It would be more appropriate to more fully describe the previous computational frameworks cited in the paper. The contrasting with the present work is more appropriate for the discussion section.

   – *Thank you for the suggestion. We have expanded the introduction to focus more on previous computational frameworks reported in the literature.*

2. There is a logical conflict between the stated goals of the paper in the introduction and the approach taken. Indeed, as stated, there is a pressing need in the field to analyze iPSC-derived cardiomyocyte structure. However, this is a challenging problem because the sarcomeres in these cells are often not fully formed (i.e. immature as mentioned in the introduction). However, all of the synthetic data presented in the paper assumes perfectly formed sarcomeres. Further, the images picked for the optimization/validation of this method are cherry picked as very good single cell iPSC-derived cardiomyocytes except for those presented in Figure 8-E4. However, the latter is not at a high enough resolution to judge if the reconstruction of the architecture is of good quality. In general, this is a non-trivial requirement for an automated analysis code, but the authors need to discuss how ignoring immature sarcomeres affects their results.

   – *Thank you for pointing this out. We have: (1) added an additional statement to the introduction to clarify this "sarcomere chains" vs. "sarcomeres," (2) added a statement on this as a key limitations in the Discussion, and (3) included an appendix example (S3 Text) that includes lower resolution and thus less clearly defined sarcomeres. A high resolution version of E4 is included on our GitHub page:* `https://github.com/elejeune11/Sarc-Graph/tree/main/ALL_MOVIES_`

RAW/experimental_data. *We have drawn attention to this in the text. And, in S1 Text and S2 Text we challenge the segmentation component of Sarc-Graph on multiple different datasets.*

3. The other issue with iPSC derived cardiomyocytes is that the sarcomeres will often deform out of sync with each other. It is not clear how the metrics described in Eq. 1-4 would be affected by non-synchronous dynamics. It is imperative that such motion be considered theoretically in order to interpret experimental results.

   – *Thank you for drawing attention to this. We have added additional clarification to the text in response, highlighted in red. In addition, we note that synthetic data examples S3, S4, and S5 contain asynchronous ground truth deformation.*

4. As this is a paper targeted at the computational/image analysis community, there needs to be more context in the description of the experimental results. Are these typical cells? Are these the best possible cells achieved with the given technique? Would the image analysis work on iPSC-derived cardiac tissues that were not well formed? Basically the reader needs more context to judge how challenged the code was.

   – *Thank you for pointing out that this was not clear. It is our sincere goal to create a software that is broadly applicable and not just to the highest quality possible images. In response to the reviewer's feedback: (1) These are typical cells when the protocol described in Sharma et al. 2018 is used, (2) we have selected a few representative cells that are not strictly "best possible" cells (e.g., E4 is in a monolayer with difficult to resolve features, and E5 contains faint z-discs and exhibits very small motion), and (3) In our experience, the code will function properly if sarcomere chains are poorly formed (e.g., nonlinear, quite short) but will not function properly if it is not possible to resolve individual z-discs (or an alternative striated feature). In addition to providing new synthetic data analysis in Supplementary Text 3 and Supplementary Text 4, we have also performed segmentation on real cell data from Sutcliffe et al 2018 in Supplementary Text 1 and from Pasqualini et al. in Supplementary Text 2. Of course, there will be limitations – particularly (as the reviewer mentioned) with detecting sarcomeres that are extremely poorly formed. We have updated the text to reflect this, including a more detailed discussion of limitations in the revised version.*

5. Additionally, the brevity of the experimental section was a bit concerning if any of the data was new. If all of the experimental data has already been published previously, that needs to be more clearly stated in each row of Table 1 where the published data can be found. As I read it, there need to be more details for E3-E5.

   – *Thank you for pointing this out. We note that there was no experimental novelty associated with this paper – in the revised version we have made the source for additional information clearer. This includes a reference to a paper in the journal "Current Protocols in Human Genetics" which provides substantial additional detail.*

6. Generally, the paper organization needs to be improved. Some of the descriptions in the "Materials and Methods" section should be moved into the "Results" Section. In particular, "Computing average deformation" and "Comparing average deformation to average sarcomere shortening and orientational order parameter" would be better suited for the "Results" section.

   – *Thank you for the suggestion. We respectfully disagree and would prefer to keep the introduction of new concepts and equations to the methods section. If the reviewer and editor are still concerned after the next round of revisions we would be happy to reconsider. We sincerely hope that the revised version of the manuscript is a substantial improvement.*

7. There looks to be a huge loss in sarcomeres that are partially tracked vs fully tracked (Fig. 7-8). Additionally, there is a huge loss in sarcomeres between the segmented image and the partially tracked image. Based on Figure 7, it appears that sarcomeres from parallel myofibrils that are aligned or in register, are treated as one sarcomere and are consequently not partially or fully tracked. This needs to be explained and put into context of applicability of this code.

   – *Thank you for drawing attention to this. We have now covered this point in the further discussion of limitations highlighted in red. Critically, sarcomere width is also a tracked parameter so it would be straightforward to define the weighted average of parameters where sarcomeres with larger widths could be weighted more heavily. Thought we do not implement this in the current version of our code, it would be a straightforward post processing step to add if necessary in the future.*

8. It would also improve the paper to directly compare the segmentation and metrics reported by "Sarc-Graph" with those done by other software (Pasqualini et al., 2015; "SarcOptiM" by Pasqualin et al., 2016; "SarcOmere Texture Analysis" Sutcliffe et al., 2018; "SarcTrack" by Toepher et al., 2019). In particular, the segmentation and tracking should be directly compared with "SarcTrack" by Toepher et al., 2019. In that work they provide metrics that quantify contractility and sarcomere structure, such as duration of contraction and relaxation, sarcomere shortening, and sarcomere length. Will computing the sarcomere shortening with their software yield the same results as Sarc-Graph? Why or why not? Further, there should be more discussion on how the contractility metrics reported by Sarc-Graph (Favg, Ciso, C——) relate to other contractility metrics or any methods that directly measure contractility (Traction Force Microscopy).

   – *Thank you for these suggestions, in response we have added substantially to our paper:*

      A. **Pasqualini et al. 2015***: Multiple metrics are defined to quantify static images of cardiomyocytes at multiple levels of maturity. Then, these metrics are used to construct a machine learning based cell scoring algorithm. Unfortunately, the code relevant to this paper is not available online. However, there was a dataset of cell images available. With this dataset, we were able to demonstrate that our segmentation code could be used for machine learning based image classification and thus structural phenotyping. This work is discussed primarily in the Appendix and mentioned in the discussion of our new draft.*

      B. **"SarCoptiM" Pasqualin et al. 2016***: Fast Fourier Transform (FFT) based analysis that relies on manually selecting a cell axis and measuring the average length of the sarcomere chains. We do not directly compare Sarc-Graph to SarcOptiM because SarcOptiM (1) relies on a manual input step thus we are concerned about bias in comparison and (2) relies on alignment and contractile synchrony in sarcomere chains and thus has limited relevance to our results. However, we have added additional discussion of SarCoptiM to the introduction of this paper.*

      C. **"SOTA" Sutcliffe et al. 2018***: Multiple metrics based on Haralick texture features are defined and used to compare between groups. Critically, there is a metric that represents "sarcomere length" which is potentially analogous to the metrics that we propose in this work. Though we were unable to get the code to run (see Fig. 1) we were able to download the relevant dataset and compare the performance of Sarc-Graph on the images shown in Fig. 4 and Fig. 6 of Sutcliffe et al. 2018. Overall, we found non-trivial discrepancies between Sarc-Graph metrics and SOTA metrics. This is explored in more detail in the Appendix.*

      D. **"SarcTrack" Toepher et al. 2019***: In the Appendix, we now include a direct comparison between outputs from our code and outputs from SarcTrack in processing the synthetic data generated for the SarcTrack paper. In general, there is a good agreement between the two codes and good agreement between Sarc-Graph and the known ground truth. These results are presented in the Appendix. We note briefly that the synthetic data generated for SarcTrack is likely "best case scenario" data for the SarcTrack code because the underlying time series of each sarcomere*

is a perfect sawtooth function. In general, SarcTrack is unable to successfully process many of the synthetic data examples that we present in our manuscript. However, because SarcTrack is (1) subject to further development and (2) relies on expertise for input parameter selection we do not make any strong claims based on this failure.

E. **TFM***: We have noted TFM in the discussion of our manuscript. Thank you for the suggestion.*

9. Further to the above point, it is unclear how the authors differentiate in their measure of contractility between the stress generated by shortening of the sarcomeres, the possible bending of the sarcomeres as the myofibril is deforming, and the resultant applied force. This is a very challenging problem as the material properties of the myofibril are non-simplistic. As a result, the metrics proposed in this work are at best an indirect measure of contractility. As such, it is important that the authors clearly state the actual physical meaning of the proposed metrics. Without such a clear-cut explanation, these would be greatly misused and misinterpreted in the field.

   – *Thank you for pointing out that this was unclear. In the revised version of the manuscript we make it clearer that our metrics do not directly measure force in any way. Instead, these metrics measure the collective kinematic behavior of a network of sarcomere chains. We make sure to point this out in the discussion of our revised manuscript.*

10. The Discussion section lacks a serious comparison to other works, existing metrics, and a in depth discussion of how the metrics should be applied AND the situations where the metrics should not be used.

    – *Thank you for pointing this out. We have now made substantial direct comparison to other works (see our response to point 8) and include the outcomes of this comparison in our manuscript. We also now directly point out the limitations of our work in the discussion.*

11. Further, the discussion section should be expanded to explain how this new method contributes to a deeper understanding of the biology of the IPSC-derived cardiomyocytes.

    – *Thank you for pointing out that this was unclear. We have expanded on this point in the revised version of the manuscript – we note that this is a major area for future work.*

12. For this work to be impactful better documentation is needed. In particular, setup.py / requirements.txt file should be added and it should include better instructions on how to set up the environment, including package versions. The package links included in the README.md were not sufficient for this reviewer to be able to run Sarc-Graph. This is an important step to be able to properly review this manuscript.

    – *Thank you for pointing this out. We have added a "`requirements.txt`" file to the GitHub page and instructions for setting up a virtual environment. If this is not sufficient to run the code please let us know – it is our intention to create a software that can be used by others.*

Minor:

1. Z-discs is spelled "z-disk" in Fig 3c.

   – *Fixed – thank you for pointing this out.*

2. Experimental movies/images should have scale bars.

   – *We have now added scale bars to Fig 7, 8, and 9 – thank you for pointing this out.*

3. It would be helpful if the sub-figures actually had labels that were referenced specifically in the text.

   – *Thank you – we have gone through our manuscript again and made some small tweaks to improve clarity.*

In summary, while this paper presents a possible solution to a pressing problem in the field, its results were not presented in a manner that would allow for in depth analysis and interpretation of the applicability of the code to a wide range of experimental data.

```
Dot indexing is not supported for variables of this type.

Error in generatePlots (line 31)
[eX,eY] =
beeswarmPoints([eCM.all.Area_um2],xspacing,(ylimits(2)-ylimits(1))/nBins,ylimits);

Error in master (line 166)
generatePlots;
```

Figure 1: Error message in MATLAB R2019a when running SOTA

Thank you to the Reviewer for taking the time to consider the revised version of our manuscript. We very much appreciate the specific suggestions for how to improve our work.

Referee 2 Comments & Response to Reviewer

"Sarc-Graph: Automated segmentation, tracking, and analysis of sarcomeres in hiPSC-derived cardiomyocytes" is a particularly interesting manuscript regarding an automated segmentation and mechanical analysis procedure for sarcomeres in cardiomyocytes. Broadly, the image processing procedures are well-executed and characterized, and I am appreciative of the authors' attention to detail in their assessment of the robustness of their algorithms and analysis. There are a few areas where the paper can be shored up and perhaps made more intuitive to a broad (i.e. non-cardiomyocyte-related) audience, but I'm quite supportive of the work.
I'll begin with some broad points, and follow with specific line items.

A1) Some more detail about the structure and optical imaging of sarcomeres would be welcome. Perhaps a schematic with some definitions would clarify to the uninformed what a sarcomere, z-disc, etc. are considered in terms of lengths, bounds, etc.

- *Thank you for the suggestion! We have added a schematic to Figure 1 and some brief additional explanation to the text.*

A2) There are a variety of typos, one of the more common ones was permuting the 'u' and 'o' in fluorescent. It'd be worth an additional check to catch these, some hyphenation issues, etc.

- *Thank you for the suggestion – we have run an additional check.*

A3) Regarding angular error: the pixel error from z-discs being small may have an outsized effect on the measured angular rotation between neighboring discs. Can this be approximated? What effects does this propagate to F? This would be of interest for imaging with different objectives, etc. There's allusion to this in ln.207, but even an approximate quantification would be helpful.

- *Thank you for this suggestion – we have added a supplement in the appendix – Supplemental Text 3 – to address this.*

A4) The metric of "shortening" is somewhat opaque to me, possibly just because of the $+1$ in the denominator. Why is it defined as it is, rather than e.g. simply delta-y/ymax? Why not use something conventional, like axial strain?

- *Thank you for pointing this out. We formulated the metric this way to match previous work in the literature (specifically the software SarcTrack). If one starts with $s = (L_{\max} - L_{\min})/L_{\max}$ (where L is length in pixels) and substitutes $y_{\min} = (L_{\min} - \bar{L})/\bar{L}$ and $y_{\max} = (L_{\max} - \bar{L})/\bar{L}$ manipulated to isolate $L_{\min}$ and $L_{\max}$ the 1 in the denominator shows up. We have added a line of clarification to the text which should help future readers.*

A5) Is there any physical interpretation of the structural tensor T? I'd also recommend using a different set of variables for the eigenvalues, as the eigenvectors for the stretch tensor are given as u already. Perhaps change eig(U) to lambdai (e.g. as in Holtzapfel).

- *Thank you for the suggestion – we have changed u to a (we also use λ previously). The structural tensor is useful for describing the degree and directioon of fiber alignment. We have added a brief additional note to the text.*

A6) The use of the Jacobian of the deformation gradient tensor seems incorrect to me. J is conventionally det(F), and is defined as the norm of F in ln.397. Regarding Ciso as well, it seems to me that the square root would be true for an assumed 2D setup, where in the 3D version it should be a cube root instead.

- *Thank you for pointing this out – we have clarified in the revised version.*

A7) I just want to reiterate that the tests for robustness are really well done, and I especially support the use of the viridis map and matplotlib package for accessibility. Nice work.

- *Thank you! We hope that the additional examples added to the revised version further strengthen the case for using our software.*

Minor comments by line:

9. Unsure what "more pressing" is being compared to

   - *We have clarified. Thank you.*

25. Shown

   - *Fixed, thank you.*

T1. What direction is vertical?

   - *Clarified – thank you.*

F3. legend. Is the "raw" 2D input image experimental or synthetic?

   - *Synthetic – now clarified. Thank you.*

122. Fluctuating

   - *Fixed thank you.*

139. Its

   - *Fixed thank you.*

176. What's considered a particle?

   - *Clarified – thank you.*

249. Sarcomeres is pluralized, possibly shouldn't be

   - *Fixed – thank you.*

362. Two ns in on

   - *Fixed – thank you.*

Thank you for taking the time to review our manuscript and for providing detailed comments! We appreciate it.
