# Supplementary material for: Sarc-Graph: Automated segmentation, tracking, and analysis of sarcomeres in hiPSC-derived cardiomyocytes
Source: PLoS Comput Biol. 2021 Oct 6;17(10):e1009443. doi: 10.1371/journal.pcbi.1009443 (PMC8523047; doi:10.1371/journal.pcbi.1009443)
Supplement: Attachment — Submitted filename: rebuttal.pdf [file pcbi.1009443.s011.pdf]

PLOS Computational Biology (PCOMPBIOL-D-21-00225) - [EMID:6864f4224b90f156]

Response to Reviewers

Sarc-Graph: Automated segmentation, tracking, and analysis of sarcomeres in hiPSC-derived cardiomyocytes

---

We would like to begin by thanking the Editor and the Reviewers for taking the time to review both our original manuscript and our revised submission. We very much appreciate their feedback and believe that the manuscript is much stronger because of it. Our response to the second round of revisions is as follows.

Referee 1 Comments

The authors have addressed almost all of my concerns. However, I was still unable to run the code. It give the following error:

Traceback (most recent call last):
File "run_code.py", line 1, in
import file_pre_processing as fpp
File "mntcUsersAdminDocumentsPythonSarc-Graphfile_pre_processing.py", line 1, in import av
ModuleNotFoundError: No module named 'av'

It is essential that the code can be run by the readers for this work to have the desired impact.

Response to Referee 1

Thank you so much for reviewing our manuscript and attempting to run our code. We have tested our software in a virtual conda environment now on Mac, Linux, and Windows devices and it works in all three cases provided that Anaconda is set up properly to help with controlling the python version and package installation. These instructions (including two extra tips for Windows which we had not tested previously) are in our public README.md file `https://github.com/elejeune11/Sarc-Graph/blob/main/README.md`. We are able to reproduce the Reviewer's error when we try and run out code without first following the Python package installation instructions. The reviewer is correct in that without the Python packages specified in "requirements.txt" installed the code will not run. That being said, if the reviewer follows the instructions and is unable to run the code, we are more than happy to revisit this issue. We would genuinely like to ensure that our code is accessible and welcome any additional suggestions or feedback.

Referee 2 Comments

I'm grateful for your attention to detail in the improved version of this manuscript, and enthusiastically support its acceptance to PLoS Comp Biol.

Response to Referee 2

Thank you for taking the time to review our manuscript. We appreciate it.
